# Comparative Network Pharmacology and Molecular Docking Analysis of Ursodeoxycholic Acid and Tauroursodeoxycholic Acid in Alzheimer’s Disease-Associated Molecular Networks

**DOI:** 10.3390/ph19101512

**Published:** 2026-09-23

**Authors:** Bo Zhou, Yu Zhong, Xin Meng, Baoyue Xu, Liwei Jia

**Affiliations:** School of Pharmacy, Heilongjiang University of Chinese Medicine, No. 24 Heping Road, Harbin 150040, China

**Keywords:** Alzheimer disease, ursodeoxycholic acid, tauroursodeoxycholic acid, bile acids and salts, network pharmacology, molecular docking, neuroprotection

## Abstract

**Background/Objectives:** Alzheimer’s disease (AD) is a multifactorial neurodegenerative disorder characterized by amyloid- and tau-related pathology, neuroinflammation, metabolic dysregulation, and neuronal loss. Previous studies have shown the potential effects of bile acids, especially ursodeoxycholic acid (UDCA) and tauroursodeoxycholic acid (TUDCA), on neurodegenerative diseases. However, whether UDCA and TUDCA exhibit distinct molecular interaction patterns within an Alzheimer’s disease-associated biological context remains unclear. This study aims to compare the relevant targets, enrichment pathways, and docking profiles predicted by UDCA and TUDCA using the computer network pharmacology framework. **Methods:** The study combined analysis of networks relating to drug action with methods examining how structures bind. We predicted potential targets of UDCA and TUDCA by using public databases and intersected with AD-related genes. Protein–protein interaction network construction, Gene Ontology and Kyoto Encyclopedia of Genes and Genomes enrichment analyses, and integrated compound–target–pathway network analysis were performed. Key targets were further evaluated by molecular docking to characterize predicted binding patterns and ligand–target interaction profiles. **Results:** UDCA and TUDCA share 98 and 103 intersecting genes with AD, respectively, with significant overlapping targets. Functional enrichment analysis showed that UDCA-related targets were significantly enriched in nuclear receptor activity, transcriptional regulation, and metabolic pathways. TUDCA-related targets are more significantly enriched in cell survival, kinase activity and neural activity ligand–receptor interactions. Molecular docking analyses provided target-specific structural information, while same-target docking using GPBAR1 and MAPK14 further revealed ligand-dependent differences in predicted interaction patterns under identical receptor conditions. Results from approaches examining systems and structure both indicate that ursodeoxycholic acid and tauroursodeoxycholic acid show profiles of action that work together. UDCA was associated with predicted upstream regulatory and metabolic-related target profiles, whereas TUDCA showed greater association with intracellular signaling, apoptosis-related, and neuronal survival-related pathways. **Conclusions:** The two bile acids showed predicted associations with pathways related to cell death regulation, inflammation, and metabolism. These findings provide a methodological basis and testable hypotheses for future experimental validation, but they should not be interpreted as direct evidence of therapeutic efficacy in Alzheimer’s disease.

## 1. Introduction

Alzheimer’s disease (AD) is a progressive neurodegenerative disorder and the leading cause of dementia worldwide [1,2,3]. Its pathology is complex and extends beyond amyloid-β deposition and tau abnormalities. Neuroinflammation, oxidative stress, mitochondrial dysfunction, metabolic disturbance, and progressive neuronal loss all contribute to disease onset and progression [4,5,6,7]. This biological complexity partly explains why single-target strategies have shown limited success in modifying the course of AD. There is still a need for agents that can act on multiple pathogenic processes at the same time.

In recent years, the evidence of relevant research on ursodeoxycholic acid and tauroursodeoxycholic acid as representative endogenous bile acid derivatives has been rapidly increasing [8,9]. UDCA is an endogenous secondary bile acid that has long been used in hepatobiliary disease, while TUDCA is its taurine-conjugated derivative [10]. Beyond their established hepatic applications, both compounds have shown anti-apoptotic, anti-oxidative, anti-inflammatory, and mitochondrial protective effects in the nervous system [11,12]. A large body of evidence over the past decade suggests that bile acids are an important class of endogenous signaling molecules. Contrary to the traditional view, a growing body of research shows that bile acids are not only metabolic end products synthesized by the liver and used to emulsify fats to aid digestion and absorption, but also have the ability to affect brain function through the gut–liver–brain axis and systemic signaling pathways [11,12]. Studies in other neurodegenerative settings have also shown that TUDCA can stabilize mitochondrial membranes, reduce oxidative stress, relieve endoplasmic reticulum stress, and suppress apoptosis [12,13,14,15,16,17]. UDCA has likewise shown protective effects in models of neurodegeneration, including improvement in mitochondrial function, regulation of autophagy, and attenuation of oxidative injury [16]. In conclusion, given the multifactorial pathogenic background of Alzheimer’s disease, both bile acids may be closely related to it.

However, current evidence has mostly examined UDCA and TUDCA separately. Direct comparisons remain limited, particularly in the context of AD. This is an important gap. Although the two compounds are structurally related, they are not necessarily pharmacologically interchangeable. Prior work in retinal degeneration models suggested that UDCA and TUDCA can produce distinct transcriptional responses, with TUDCA affecting a much broader set of genes than UDCA [18]. The extent to which molecular targets and pathways that these compounds affect show overlap or difference in the context of disease pathology is unclear. In a study of retinal degeneration, TUDCA regulated an order of magnitude higher than UDCA, and only a small proportion of genes were co-regulated. In addition, the regulatory mode of TUDCA in endoplasmic reticulum stress and neurological development-related pathways is significantly different from UDCA [19]. These studies show that although UDCA and TUDCA are both endogenous hydrophilic bile acids and have partially overlapping biological effects, they show significant functional differentiation in molecular targets and signaling pathway preferences. Treatment for Alzheimer’s disease may depend on metabolic regulation, inflammation control, and neuronal survival, and the mechanistic differences between these three are potentially significant.

Therefore, in order to deepen the understanding of the mechanisms of action of these two compounds and provide a scientific basis for their precise development in Alzheimer’s disease-related intervention strategies, it is necessary to make a clearer comparison between UDCA and TUDCA. Rather than treating them as a single class with similar effects, it may be more informative to define where their target networks converge and where they diverge [20]. This is especially relevant for a disease such as AD, in which therapeutic benefit is unlikely to depend on one pathway alone.

Alzheimer’s disease is a complex multifactorial disorder involving amyloid and tau pathology, neuroinflammation, oxidative stress, mitochondrial dysfunction, and metabolic disturbances. Increasing evidence suggests that bile acid signaling participates in several biological processes relevant to AD-associated pathology, including cellular stress responses, inflammatory regulation, and metabolic homeostasis. Therefore, investigating UDCA and TUDCA within an AD-associated molecular framework may provide insights into how structurally related bile acids differentially interact with disease-relevant molecular networks.

It should be noted that the molecular targets identified in this study are not considered AD-specific AD-associated candidate targets, as many regulators involved in neurodegeneration are shared among multiple neurological disorders. Instead, this study focuses on the intersection between compound-associated predicted targets and AD-associated gene sets to characterize shared and divergent molecular patterns of UDCA and TUDCA under an AD-related biological context.

In this study, we performed a comparative network pharmacology analysis of UDCA and TUDCA in AD and complemented it with molecular docking. We aimed to identify AD-related targets shared by the two bile acids, define their distinct hub genes and enriched pathways, and evaluate representative compound–target interactions at the structural level. We hypothesized that UDCA and TUDCA would show partially overlapping AD-associated molecular networks, but would differ in the relative emphasis of their pharmacological actions. More specifically, UDCA was expected to be more closely associated with metabolic and nuclear receptor-related regulation, whereas TUDCA was expected to show greater representation of signaling-, apoptosis-related, and neuroactive pathway-associated targets. By clarifying these shared and distinct features, this study seeks to provide a more precise mechanistic basis for understanding the potential molecular roles of bile acids within AD-associated biological networks.

## 2. Results

### 2.1. Target Intersection Screening and Venn Diagram Analysis

In this study, we screened a total of 106 potential targets of ursodeoxycholic acid and 111 potential targets of tauroursodeoxycholic acid through target prediction (Figure 1). We then compared these predictive targets with Alzheimer’s disease-related genes obtained from the GeneCards and OMIM databases. To evaluate whether target identification was affected by the GeneCards filtering threshold, a sensitivity analysis was subsequently performed using alternative relevance score cutoffs (>0, >1, >5, and >10). To evaluate whether target identification was affected by GeneCards filtering thresholds, sensitivity analysis was performed using relevance score cutoffs of >0, >1, >5, and >10. Although increasing the threshold reduced the number of AD-associated genes, the major overlapping targets remained relatively stable. The numbers of UDCA–AD and TUDCA–AD overlapping targets were 98 and 103 at the primary threshold (>1), respectively, and remained 61 and 77 even under the most stringent threshold (>10). All major hub targets used for subsequent network interpretation and docking prioritization were consistently retained across all tested thresholds (Supplementary Tables S10–S12).

Additional comparison shows that ursodeoxycholic acid and tauroursodeoxycholic acid present considerable overlap in targets. This overlap includes main genes relating to Alzheimer’s disease such as BCL2, which functions in regulation of cell death, HMGCR, which is an important factor in the process that produces cholesterol, and OPRM1, which is a receptor type. These targets that appear in both compounds suggest that the two compounds may function through similar processes in affecting Alzheimer’s disease features.

The analysis also reveals targets that are specific to individual compounds. Ursodeoxycholic acid shows specific targets such as ESR1 and PPARA, which are receptors that belong to a particular group and may function in regulation of gene activity and processes relating to metabolism. In contrast, tauroursodeoxycholic acid shows specific targets including DRD2 and SLC6A4, which indicates that this compound may function more in processes that involve signals between nerve cells. These differences between the compounds suggest that ursodeoxycholic acid and tauroursodeoxycholic acid may affect Alzheimer’s disease through different molecular processes.

### 2.2. Independent Support Evaluation of Predicted Targets

To further evaluate the external support of computationally predicted targets, SwissTargetPrediction-derived targets of UDCA and TUDCA were compared with experimentally supported drug–target associations obtained from DrugBank and ChEMBL databases. After standardization and filtering of ChEMBL records to human single-protein targets, several predicted targets showed independent pharmacological support (Supplementary Tables S13 and S14).

For UDCA, nine predicted targets were supported by ChEMBL-associated experimental records, including GPBAR1, NR1H4, ESR1, VDR, and several transporter- or receptor-related proteins. Among these targets, NR1H4 (farnesoid X receptor, FXR) was additionally recorded in DrugBank, providing consistent support from independent computational and curated pharmacological resources. GPBAR1, a recognized bile acid-responsive receptor, was also supported by ChEMBL evidence and was retained as a shared target for subsequent comparative docking and molecular dynamics analysis.

For TUDCA, experimentally supported overlap was observed mainly for bile acid-related receptors, including GPBAR1 and NR1H4. These findings indicate that although database-based target prediction generates a broader candidate target landscape, several biologically relevant targets identified by computational prediction are supported by independent pharmacological evidence.

Collectively, this analysis provides additional confidence for subsequent network prioritization and structural analyses while emphasizing that computational target prediction should be interpreted as hypothesis-generating rather than a complete representation of experimentally confirmed pharmacological interactions.

### 2.3. GO Functional Enrichment Analysis

Based on UDCA-AD and TUDCA-AD related datasets, we performed functional analysis of the screened targets. As shown in Figure 2 and Supplementary Table S1, the two compounds displayed clearly different enrichment profiles.

UDCA-associated targets were mainly enriched in nuclear receptor activity, ligand-activated transcription factor activity, steroid binding, and related transcriptional regulatory functions, indicating a profile centered on receptor-dependent regulation and metabolic responsiveness. TUDCA-associated targets were more strongly enriched in kinase-related and receptor-mediated signaling functions, including protein serine/threonine kinase activity, phosphatase binding, and peptide receptor activity.

Overall, these findings indicate that UDCA is more strongly linked to nuclear receptor-dependent regulatory functions, whereas TUDCA is more closely related to intracellular signaling and rapid receptor-mediated responses.

### 2.4. KEGG Pathway Enrichment Analysis

KEGG enrichment analysis further highlighted both shared and distinct pathway preferences between UDCA and TUDCA (Figure 3; Supplementary Table S2).

For UDCA, enriched pathways were mainly related to neuroactive ligand–receptor interaction, hormone-associated signaling, and selected receptor-regulatory pathways, including PPAR, estrogen, and thyroid hormone signaling. Additional terms suggested links to inflammatory regulation, lipid-related signaling, and synaptic or clearance-related processes. For TUDCA, the dominant pathways were more strongly centered on neuroactive signaling, PI3K–Akt, cAMP, neurotrophin, and inflammation-related pathways, indicating a closer association with survival signaling, stress responses, and broader receptor-mediated networks.

Comparative analysis indicated that both bile acids converged on neuroactive ligand-responsive signaling, but differed in their dominant pathway preferences. UDCA showed a stronger association with receptor-linked and hormone-related regulation, whereas TUDCA was more strongly connected to intracellular survival and inflammatory signaling. These results support the view that the two bile acids may share partial mechanistic overlap in AD while still differing in their major pharmacological emphases.

### 2.5. PPI Network Analysis and Identification of Hub Targets

The targets that overlap between UDCA and AD and between TUDCA and AD were separately put into the STRING database to develop networks showing protein to protein interaction (Figure 4 and Figure 5). Analysis examining the structure revealed differences in structure and in measures of central position between the two networks.

In the network for UDCA targets, hub targets were identified based on degree centrality, with the top 20% of nodes retained for further prioritization (Figure 4). SRC showed the highest degree centrality (degree = 44), followed by ESR1 and EGFR (degree = 32 for both), PPARA (degree = 25), and RXRA (degree = 21). CYP19A1, RARA, and NR0B2 showed the same degree value (degree = 17). Among these tied nodes, CYP19A1 showed higher betweenness and closeness centrality than RARA and NR0B2 and was therefore prioritized as the sixth representative target for subsequent molecular docking. Accordingly, SRC, ESR1, EGFR, PPARA, RXRA, and CYP19A1 were selected for structural analysis. The network also included members of the nuclear receptor group such as RXRA (receptor alpha for retinoid X), RARA (receptor alpha for retinoic acid), and AR (receptor for androgen), and also included factors that act to limit production of gene products such as NR0B2 (SHP), which suggests that UDCA may produce effects that regulate production of gene products in ways that are broad. In addition, some targets that participate in transmission of signals between nerve cells were found, including GRM1/2/5 (receptors for glutamate that operate through processes inside cells), OPRM1 (receptor for opioid acting at mu position), and CHRNA7 (subunit α7 of receptor for acetylcholine of nicotinic type). These targets relating to nerve cells showed relatively lower measures of central position by degree and did not form major hubs in the network.

The TUDCA-related network contained 103 nodes and showed a different centrality pattern (Figure 5). In the TUDCA-associated PPI network, 22 targets were retained within the top 20% according to degree centrality (Figure 5). Among these hub targets, BCL2 showed the highest degree (degree = 70), followed by PTGS2 and MTOR (degree = 62 for both), JAK2 and KIT (degree = 50 for both), and PIK3CA (degree = 48). These six proteins therefore represented the highest-ranked targets according to degree centrality and were selected for subsequent molecular docking. These findings suggest that TUDCA may preferentially influence pathways associated with intracellular signal transduction and stress-response regulation.

The TUDCA network also included several proteins involved in neurotransmitter-related processes, such as DRD2, SLC6A3, SLC6A4, and ACHE. These proteins were connected either directly or indirectly through intermediate nodes, suggesting that TUDCA may also be linked to dopaminergic, serotonergic, and cholinergic signaling systems. In addition, AD-related proteins such as PSEN1 and APOE were present in the network. Although they were not among the top-ranked hubs, their inclusion supports the relevance of the TUDCA-associated target set to AD pathology.

The networks for UDCA and TUDCA show features that are similar and features that differ. Analysis indicates that certain main elements appear in both networks. AKT1 represents an element that occurs in the network for UDCA and in the network for TUDCA. This suggests that the two compounds relate to pathways involving survival of cells and processes involving use of energy. The relationship likely occurs through signaling that involves AKT1. However, the network for UDCA contains particular elements that relate to receptors in the cell nucleus. ESR1 and PPARA represent examples of such elements. This finding supports the view that UDCA functions through processes that involve control of gene expression and processes that maintain balance in use of energy. In contrast, the TUDCA network showed greater prominence of apoptosis-, inflammation-, and signaling-related nodes, including BCL2 and PTGS2. Overall, these results are in line with the GO and KEGG analyses and further support the view that UDCA is more closely associated with nuclear receptor-linked and metabolic regulatory networks, whereas TUDCA shows a stronger association with cell survival, inflammatory signaling, and neuronal signaling-related pathways.

### 2.6. Integrated Network: Compound–Target–Pathway–Disease Associations

To visualize the relationships among UDCA, TUDCA in Alzheimer’s disease, a network structure that combines these compounds, the main KEGG pathways showing significance, and the disease representation appears in Figure 6. The network showed partial overlap between the two bile acids at the level of neuroactive signaling and receptor-mediated pathways, while also indicating different dominant pathway preferences. UDCA was more closely connected to receptor-regulatory and hormone-associated pathways, whereas TUDCA showed stronger links to survival signaling, inflammatory regulation, and broader stress-response pathways. These network features were consistent with the enrichment analyses and support the idea that UDCA and TUDCA may act through partially overlapping but non-identical mechanisms in AD.

### 2.7. Validation of the Molecular Docking Protocol

Native-ligand redocking was performed for all 14 protein structures to evaluate the reproducibility of the docking protocol. Twelve of the fourteen receptor–ligand systems reproduced the corresponding crystallographic ligand conformations with RMSD values below 2.0 Å, with values ranging from 0.000 to 1.971 Å (Supplementary Table S7). These results indicate satisfactory reproduction of the experimentally observed binding poses for the majority of the selected protein structures.

Two systems showed RMSD values above the predefined 2.0 Å criterion. The redocked native ligand of JAK2 (PDB ID: 3UGC) showed an RMSD of 2.169 Å, whereas the MTOR structure (PDB ID: 4DRI) showed an RMSD of 3.872 Å. Therefore, the docking poses obtained for these two receptor systems, particularly MTOR, were interpreted with additional caution. Overall, the redocking analysis supported the general applicability of the docking protocol while also identifying receptor-specific differences in pose-reproduction performance.

### 2.8. UDCA (CYP19A1, EGFR, ESR1, PPARA, RXRA, SRC)

Molecular docking was performed to characterize the predicted binding modes of UDCA with six prioritized AD-related targets, including CYP19A1, EGFR, ESR1, PPARA, RXRA, and SRC. The predicted binding energies ranged from −8.1 to −7.0 kcal/mol (Table 1; Figure 7).

PPARA yielded a predicted docking score of −8.1 kcal/mol, with the selected pose displaying a polar interaction involving Ile420. CYP19A1 showed a predicted binding energy of −7.9 kcal/mol, with interactions involving Ala306, Thr310, and Arg115. For ESR1, the predicted binding energy was −7.1 kcal/mol, and the selected pose showed interactions with Tyr459 and His476. UDCA docking with RXRA yielded a predicted binding energy of −7.0 kcal/mol, with interactions involving Met454, Arg302, Ala303, and Asn377.

For the kinase-related targets, SRC showed a predicted binding energy of −7.2 kcal/mol and an interaction involving Met91. EGFR showed a predicted binding energy of −7.0 kcal/mol, with interactions involving Asp163 and Met101. Collectively, these docking results identified computationally favorable binding poses of UDCA within the selected receptor pockets. The observed interactions with PPARA, ESR1, and RXRA were broadly consistent with the nuclear receptor-related features identified in the preceding network and enrichment analyses; however, the docking results alone do not establish functional regulation of these proteins. Because these docking calculations involved different receptor proteins and binding environments, their scores were reported descriptively and were not interpreted as direct measures of relative affinity across targets.

### 2.9. TUDCA (BCL2, JAK2, KIT, MTOR, PIK3CA, PTGS2)

Molecular docking was performed to examine the predicted binding modes of TUDCA with six prioritized AD-related targets, including BCL2, JAK2, KIT, MTOR, PIK3CA, and PTGS2. The predicted binding energies ranged from −10.3 to −7.4 kcal/mol across the evaluated receptor structures (Table 2; Figure 8).

PIK3CA yielded a predicted docking score of −10.3 kcal/mol, with the selected pose displaying interactions involving Asn170, Arg662, and Cys838. JAK2 showed a predicted binding energy of −9.3 kcal/mol, with interactions involving His974 and Asp994. KIT yielded a predicted binding energy of −8.5 kcal/mol, with interactions involving Ile167 and Arg169.

For MTOR, the predicted binding energy was −7.7 kcal/mol, with an interaction involving Ser2035. BCL2 showed a predicted binding energy of −7.4 kcal/mol, with contacts involving Tyr180, Arg183, and His184. PTGS2 showed a predicted binding energy of −7.9 kcal/mol, with interactions involving Arg425, Ser143, Lys418, and Lys144. As in the UDCA analysis, docking scores obtained from different receptor proteins were not interpreted as directly comparable estimates of binding affinity.

Overall, these results indicate that TUDCA can adopt computationally favorable binding poses within the selected kinase-, signaling-, apoptosis-, and inflammation-related protein structures. These structural observations are broadly consistent with the enrichment of TUDCA-associated targets in intracellular signaling and cell-survival-related pathways. However, molecular docking does not determine whether TUDCA activates, inhibits, or otherwise functionally regulates these targets. Because the native-ligand redocking RMSD values for JAK2 and MTOR exceeded the predefined 2.0 Å criterion, the corresponding TUDCA docking poses, particularly that of MTOR, should be interpreted with additional caution.

### 2.10. Comparative Docking of UDCA and TUDCA at Shared AD-Related Targets

Since docking scores obtained from different receptor proteins are influenced by distinct binding environments and cannot be directly compared across different targets, additional same-target docking analyses were performed. GPBAR1 and MAPK14 were selected because they were shared AD-related targets of both UDCA and TUDCA and represented biologically relevant target contexts associated with bile acid signaling and AD-related inflammatory regulation (Table 3).

Under identical GPBAR1 receptor conditions, UDCA and TUDCA produced predicted docking scores of −9.4 and −9.1 kcal/mol, respectively. Both ligands occupied the same receptor binding region but displayed different predicted interaction patterns. UDCA showed interaction mainly involving Tyr89, whereas TUDCA exhibited additional contacts with residues including Ser156, Ser157, Ser247, and Tyr240.

For MAPK14, UDCA and TUDCA showed predicted docking scores of −9.3 and −8.7 kcal/mol, respectively. The two ligands adopted distinct binding orientations within the same receptor pocket. UDCA interacted with the receptor region involving Ala74, whereas TUDCA displayed interactions involving Lys76, Glu94, Asp191, and Met132.

These results indicate ligand-dependent differences in predicted interaction patterns under identical receptor conditions. However, the docking scores were interpreted only as computational indicators of relative interaction preferences within each receptor system and were not considered direct measurements of binding affinity or biological activity.

### 2.11. Molecular Dynamics Simulation of GPBAR1–UDCA and GPBAR1–TUDCA Complexes

To further characterize the dynamic stability of the shared target interaction, 100 ns molecular dynamics simulations were performed for GPBAR1 complexes with UDCA and TUDCA. The RMSD profiles of both protein backbones and ligands are shown in Figure 9. Both complexes remained structurally stable throughout the simulation period, although ligand-dependent differences in dynamic behavior were observed.

The protein backbone RMSD profiles showed that both GPBAR1–UDCA and GPBAR1–TUDCA complexes rapidly reached equilibrium during the initial simulation stage and remained stable throughout the remaining trajectory. The ligand RMSD values indicated that UDCA maintained a relatively stable position within the binding pocket, whereas TUDCA exhibited slightly greater conformational fluctuation, which may be attributed to the additional taurine-containing side chain and increased molecular flexibility.

The radius of gyration (Rg) analysis is shown in Figure 10, while SASA profiles are presented in Figure 11. Hydrogen-bond analysis was further performed to characterize ligand–receptor interactions (Figure 12). The RMSF profiles of GPBAR1 residues and bound bile acids are presented in Figure 13.. RMSF analysis demonstrated that residue fluctuations were mainly localized in flexible loop regions rather than the predicted ligand-binding region, suggesting that ligand binding did not induce substantial structural destabilization of GPBAR1. The radius of gyration (Rg) analysis is shown in Figure 10, while SASA profiles are presented in Figure 11. The radius of gyration values remained relatively constant during the simulation, indicating that both ligand-bound systems maintained comparable protein compactness. Similarly, SASA profiles showed no significant drift, further supporting the structural stability of both complexes.

Hydrogen-bond analysis revealed persistent polar interactions between GPBAR1 and both bile acids during the simulation. Dynamic hydrogen-bond profiles between GPBAR1 and the two bile acids are shown in Figure 12. The GPBAR1–UDCA complex maintained approximately 5–6 hydrogen bonds throughout most of the trajectory, whereas TUDCA displayed a distinct interaction pattern associated with its additional taurine moiety. These results suggest that taurine conjugation may modify the hydrogen-bonding network and ligand recognition mode within the GPBAR1 binding environment.

The MM/GBSA binding free energy trajectories are shown in Figure 14. MM/GBSA calculations further supported favorable binding between both bile acids and GPBAR1. The calculated average binding free energies were −59.94 kcal/mol for UDCA and approximately −66.92 kcal/mol for TUDCA, indicating that TUDCA exhibited a more favorable predicted binding energy under the simulated conditions. Although these values should not be interpreted as experimental binding affinities, the energetic differences suggest that the taurine modification may contribute additional stabilizing interactions within the receptor environment.

MD simulations supported that both UDCA and TUDCA could form stable complexes with GPBAR1, while their interaction dynamics were influenced by structural differences between the two bile acids. Compared with UDCA, TUDCA showed slightly increased ligand flexibility but maintained stronger predicted binding energetics, providing a structural explanation for the ligand-dependent interaction differences observed in the same-target docking analysis.

### 2.12. Predicted Physicochemical Properties and ADMET Profiles

To provide additional computational characterization, physicochemical properties and ADMET-related parameters of UDCA and TUDCA were predicted using ADMETlab 3.0. The comparative radar visualization of selected physicochemical descriptors is shown in Figure 15. These results were interpreted as model-based estimations rather than experimental pharmacokinetic measurements. UDCA showed lower molecular weight and TPSA values compared with TUDCA, reflecting the structural impact of taurine conjugation. Differences were also observed in predicted absorption-related and physicochemical descriptors; however, these parameters only describe computationally predicted molecular properties and do not directly indicate superior pharmacokinetic performance in vivo.

With respect to metabolism-related endpoints, both compounds were computationally predicted to have relatively low inhibitory potential toward the major CYP isoforms evaluated, including CYP1A2, CYP2C9, CYP2C19, CYP2D6, and CYP3A4. UDCA was additionally predicted to have a higher likelihood of acting as a CYP3A4 substrate. These results represent model-based estimates and do not establish actual metabolic disposition in vivo.

For the toxicity-related endpoints evaluated by ADMETlab 3.0, neither compound showed prominent predicted hERG inhibition or mutagenicity signals, whereas selected endpoints, including skin sensitization and organ-toxicity-related predictions, indicated potential liabilities that warrant experimental verification. Overall, these computational results provide a comparative description of predicted ADMET-related properties rather than experimentally measured pharmacokinetic or safety profiles.

## 3. Discussion

### 3.1. Overview of Shared and Divergent Mechanisms

The study examines the approach combining analysis of networks and analysis of structure to compare ursodeoxycholic acid and tauroursodeoxycholic acid in Alzheimer’s disease. Findings show that the two compounds affect main processes relating to disease but differ in the specific processes that the compounds affect. The first compound mainly affects processes relating to factors in the cell that regulate other processes and processes that produce substances, and the second compound affects processes that include signals within cells and processes relating to signals between cells [18]. The two compounds show similar effects that provide protection relating to cells in the brain, and these effects include effects on processes relating to cell death and effects on processes relating to responses in tissues. The structural analysis further suggested differences in the predicted protein-interaction profiles of the two compounds, consistent with their distinct network and pathway associations. These differences indicate mechanistic divergence but do not establish additive or complementary pharmacological effects [11,18].

### 3.2. Interpretation of PPI-Prioritized Docking Results

Molecular docking analysis identified computationally plausible interaction modes between UDCA and nuclear receptor-related targets, including PPARA, ESR1, and RXRA. These predicted structural interactions were consistent with the enrichment patterns observed in nuclear receptor-associated functions; however, docking results alone cannot establish target preference or functional regulation [21,22]. In GO analysis, UDCA-related targets were significantly enriched in nuclear receptor activity, ligand-activated transcription factor activity, and steroid binding function [22]. In the corrected KEGG pathway analysis, UDCA was also significantly enriched in hormone-related pathways such as PPAR signaling pathway, estrogen signaling pathway, thyroid hormone signaling pathway, and neuroactive ligand–receptor interaction pathway.

These findings suggest that the pharmacological characteristics of UDCA are not limited to simple metabolic effects, but are integrated with nuclear receptor-mediated regulation by ligand-responsive signaling systems. The coexistence of chronic metabolic disorders, inflammatory imbalances, synaptic dysfunction and impaired adaptive regulation in the pathogenesis of Alzheimer’s disease makes this mechanism of great significance in the AD-associated molecular regulation [23]. The predicted interactions between UDCA and PPARA, RXRA, and ESR1 provide structural hypotheses supporting further investigation of nuclear receptor-related mechanisms in AD-associated biological processes [24].

These findings suggest that UDCA-associated targets may be involved in biological processes related to homeostasis maintenance, including mitochondrial function, endoplasmic reticulum stress, autophagy, and nuclear receptor-associated signaling [25,26]. This finding is helpful in distinguishing UDCA from taurine-ursodeoxycholic acid (TUDCA) at the pharmacological level and provides more specific theoretical guidance for future validation studies on its mechanism of action in Alzheimer’s disease-related experimental models.

### 3.3. Interpretation of Same-Target Comparative Docking

Because docking scores obtained from different receptor structures are affected by receptor-specific binding environments, cross-target comparisons of docking energies were avoided. To further compare UDCA and TUDCA under controlled receptor conditions, same-target docking analyses were performed using GPBAR1 and MAPK14, which were shared AD-related targets of both compounds.

In the GPBAR1 system, UDCA and TUDCA adopted different predicted binding orientations within the same receptor pocket. UDCA showed an interaction mainly involving Tyr89, whereas TUDCA formed additional predicted contacts with Ser156, Ser157, Ser247, and Tyr240. In the MAPK14 system, the two bile acids also displayed distinct predicted interaction patterns, with UDCA interacting with Ala74 and TUDCA showing contacts involving Lys76, Glu94, Asp191, and Met132.

These findings indicate that taurine conjugation may influence the structural accommodation of the bile acid scaffold within shared receptor environments. The molecular dynamics simulations further provided dynamic evidence supporting the ligand-dependent interaction patterns observed in docking analysis. Although both GPBAR1–UDCA and GPBAR1–TUDCA complexes remained stable during the 100 ns simulations, TUDCA exhibited a more favorable MM/GBSA binding energy and maintained additional polar interaction potential due to the presence of the taurine-conjugated moiety. Meanwhile, UDCA showed lower ligand fluctuation, suggesting a relatively rigid binding mode within the receptor pocket. These findings indicate that taurine conjugation does not simply enhance molecular interaction through increased polarity, but may alter the balance between ligand flexibility and receptor stabilization. Therefore, the structural difference between UDCA and TUDCA may contribute to their distinct predicted pharmacological profiles in bile acid-responsive signaling pathways. However, these docking observations should be interpreted as ligand-dependent differences in predicted molecular interactions rather than direct evidence of differential pharmacological potency, receptor activation, or functional regulation.

### 3.4. Potentially Complementary Predicted Target and Pathway Profiles

The comparative analyses identified distinct but partially overlapping predicted target and pathway profiles for UDCA and TUDCA. UDCA showed predominant associations with nuclear receptor-linked, hormone-related, and metabolic regulatory processes, whereas TUDCA was more prominently associated with kinase signaling, apoptosis-related processes, and cellular stress-response pathways. These differences suggest that the two bile acids may engage different components of AD-associated molecular networks.

From a hypothesis-generating perspective, such divergence may be described as potentially complementary at the level of predicted target and pathway coverage. However, this computational complementarity should not be interpreted as evidence of pharmacological synergy, additivity, or superior efficacy of combined UDCA and TUDCA treatment. The present study did not include combination-response experiments, target-engagement assays, or biological evaluation of the two compounds administered together. Therefore, whether their distinct molecular profiles translate into any beneficial interaction when used in combination remains to be determined experimentally [11,22,23,27].

### 3.5. Comparison with Previous Neuroprotective Studies of UDCA and TUDCA

Previous studies in other neurodegenerative diseases and retinal diseases have provided an important background for understanding the observed differences between UDCA and TUDCA [26]. Although these two bile acids have similar structures (TUDCA is the product formed by the combination of UDCA and taurine), there may be significant differences in their biological functions and mechanisms of action. In the retinal degenerative disease model, transcriptomic analysis revealed that TUDCA affected a broader set of genes compared to UDCA (Figure 16). This indicates that TUDCA has a more extensive role in regulating cellular gene expression, and the molecular network it affects may be more complex and extensive. More importantly, TUDCA shows a stronger association with endoplasmic reticulum stress (ER stress) and neurodevelopment-related responses [18]. This pattern is broadly consistent with our findings, in which TUDCA displayed greater enrichment in intracellular survival signaling, kinase-associated pathways, and anti-apoptotic targets, whereas UDCA was more closely associated with nuclear receptor-linked and metabolic regulatory processes [28].

A similar tendency can be recognized in Parkinson’s disease-related studies [29]. Previous work has shown that both bile acids can exert neuroprotection-related associations, but TUDCA has more often been linked to anti-apoptotic, mitochondrial protective, and stress-response mechanisms, whereas UDCA has also been reported to improve mitochondrial function, regulate autophagy, and attenuate oxidative injury [30]. Our comparative network and docking results fit this broader distinction reasonably well: TUDCA appears to align more closely with rapid survival signaling and apoptosis-related regulation, whereas UDCA appears to align more strongly with receptor-mediated homeostatic regulation [26].

Evidence from Huntington’s disease and related protein-misfolding disorders also supports the idea that bile acids, particularly TUDCA, may be relevant to endoplasmic reticulum stress, proteostasis disruption, and neuronal survival [11]. Although our study was focused on AD rather than Huntington’s disease, the present enrichment and docking profiles are compatible with that broader neuroprotective framework. Taken together, prior studies across retinal and neurodegenerative settings suggest that UDCA and TUDCA share a common neuroprotective background but are unlikely to be mechanistically identical [18]. This cross-disease consistency strengthens the interpretation that the divergence observed in the present AD-centered analysis is biologically plausible rather than merely methodological.

### 3.6. Translational Relevance of the Divergent Network–Docking Profiles

One of the main implications of our comparative analysis is that UDCA and TUDCA should not be regarded as pharmacologically interchangeable in the context of AD-related target networks. Although both bile acids converged on processes relevant to apoptosis, inflammation, and neuronal stress, their dominant association patterns were not identical. UDCA was more strongly connected to nuclear receptor-related and metabolic regulatory pathways, whereas TUDCA showed greater emphasis on kinase signaling, anti-apoptotic regulation, and neuroactive ligand–receptor interaction.

From a pharmacological perspective, the divergence observed here suggests that the two bile acids may differ not only in target preference, but also in the level at which they intervene in disease-related biology. UDCA was more strongly associated with pathways related to metabolic homeostasis and transcription, while TUDCA tends to regulate stress response signaling pathways related to cell survival. Compared with simply comparing molecular docking scores, this difference at the mechanistic level may be more instructive for subsequent validation studies. In clinical application scenarios, the value of comparing these two compounds is not to judge which one is better, but to explore whether they are suitable for different experimental situations or pathological states.

### 3.7. Interpretation of the Predicted Physicochemical and ADMET Differences Between UDCA and TUDCA

The in silico physicochemical and ADMET analyses indicated that taurine conjugation is associated with substantial differences between UDCA and TUDCA (Figure 15). TUDCA contains a taurine side chain linked to the C24 carboxyl group of UDCA through an amide bond, introducing additional polar and ionizable functionalities. Consistent with this structural difference, TUDCA showed a higher molecular weight and larger TPSA in the present analysis, whereas UDCA displayed different predicted profiles for several permeability- and absorption-related endpoints.

These structural differences may influence physicochemical and molecular transport-related characteristics; however, the present data do not directly demonstrate intestinal absorption, systemic exposure, tissue distribution, or blood–brain barrier penetration. In particular, Caco-2 permeability, HIA, plasma protein binding, BBB permeability, CYP-related properties, clearance, and toxicity endpoints reported here were generated computationally and should be regarded as hypothesis-generating estimates rather than experimentally measured pharmacokinetic parameters.

The predicted BBB profiles deserve particular caution in the context of AD. Although both UDCA and TUDCA have been discussed in the literature for their neuroprotective potential, the present computational analysis does not establish the extent to which either compound reaches the central nervous system in vivo. Therefore, the predicted differences identified here should primarily be used to guide subsequent pharmacokinetic and experimental validation rather than to infer comparative brain exposure or therapeutic efficacy.

### 3.8. Limitations and Future Directions

The independent target support analysis further strengthened the interpretation of the computational network results. Although SwissTargetPrediction provides a systematic approach for generating potential drug targets based on chemical similarity, such predicted interactions may include candidates that have not yet been experimentally characterized. By comparing predicted targets with DrugBank and ChEMBL records, several key bile acid-associated targets, particularly NR1H4 and GPBAR1, were supported by independent pharmacological evidence. However, the absence of database support for other predicted targets does not necessarily indicate biological irrelevance, as curated pharmacological databases are limited by experimental coverage and publication availability.

Therefore, the independent support analysis should be considered as an external evidence assessment rather than definitive target validation. The current findings integrate computational prediction, network-based prioritization, structural docking, and molecular dynamics simulation to generate testable hypotheses regarding UDCA and TUDCA in AD-associated mechanisms, while further experimental studies remain necessary to confirm individual target engagement and biological regulation.

This study has several limitations that should be acknowledged. The present conclusions are based on target prediction, enrichment analysis, ADMET estimation, and molecular docking, and therefore remain hypothesis-generating rather than experimentally validated. Docking provides a static structural snapshot and is influenced by receptor selection, pocket definition, and scoring functions. In addition, the ADMET-related parameters were derived exclusively from computational prediction and require experimental pharmacokinetic and toxicological validation before conclusions regarding systemic exposure, brain penetration, or safety can be made. Furthermore, no combination-response or target-engagement experiments were performed for UDCA and TUDCA. Consequently, the partially distinct or potentially complementary predicted target profiles identified in this study should not be interpreted as evidence of pharmacological synergy, additivity, or enhanced therapeutic efficacy of combined treatment.

At the in vitro level, Aβ-challenged neuronal models such as SH-SY5Y cells may be used as an initial validation system. Western blot analysis, together with complementary approaches such as qPCR or immunofluorescence where appropriate, could be applied to examine whether UDCA preferentially modulates targets related to nuclear receptor and metabolic regulation, such as PPARA, RXRA, and ESR1, while TUDCA more strongly affects survival- and stress-related markers such as BCL2, MTOR, and proteins within the PI3K–Akt or JAK–STAT pathways. Such experiments would help determine whether the network- and docking-based divergence identified here can be reproduced at the protein-expression and signaling-response levels.

## 4. Materials and Methods

### 4.1. Compound Selection and Target Prediction

In this study, ursodeoxycholic acid (UDCA) and tauroursodeoxycholic acid (TUDCA) were selected as the candidate compounds. The two-dimensional structures, SMILES strings, and PubChem Compound IDs (CIDs) of both compounds were retrieved from the PubChem database (https://pubchem.ncbi.nlm.nih.gov; accessed on 23 May 2025) [31]. The SMILES strings were input into the SwissTargetPrediction platform (http://www.swisstargetprediction.ch; accessed on 23 May 2025), with the species parameter set to Homo sapiens, to predict potential human protein targets. Duplicated entries and targets lacking functional annotations were excluded, and the remaining predicted targets were retained for further analysis.

### 4.2. Collection of Alzheimer’s Disease-Related Genes

AD-related genes were identified using “Alzheimer’s disease” as a keyword in the GeneCards database (https://www.genecards.org; accessed on 24 May 2025). Genes with a relevance score >1 were initially selected. Additional AD-associated genes were retrieved from the OMIM database (https://www.omim.org; accessed on 24 May 2025) [32,33]. The GeneCards and OMIM datasets were merged and deduplicated to establish the final AD-related gene set. The robustness of GeneCards-based target selection was further evaluated by sensitivity analysis using alternative relevance score thresholds (>0, >1, >5, and >10) [34].

### 4.3. Independent Target Support Evaluation

To evaluate whether computationally predicted targets were supported by independent pharmacological evidence, experimentally reported drug–target associations were retrieved from DrugBank and ChEMBL databases. DrugBank records associated with ursodeoxycholic acid (UDCA) and tauroursodeoxycholic acid (TUDCA) were collected, and target information was standardized to gene symbols using UniProt identifiers. ChEMBL-associated target records were filtered to retain human single-protein targets with experimentally reported compound–target associations. The resulting target sets were compared with SwissTargetPrediction-derived targets, and overlapping targets were considered independently supported targets. This analysis was performed to assess the external support of computational target prediction rather than to confirm all predicted interactions. Detailed target-support information is provided in Supplementary Tables S13 and S14.

### 4.4. Construction of Protein–Protein Interaction (PPI) Networks

The intersecting targets of UDCA–AD and TUDCA–AD were separately imported into the STRING database (version 11.5, https://string-db.org; accessed on 24 May 2025) [35]. The organism was set to Homo sapiens, and the minimum required interaction score was set to “high confidence” (combined score > 0.7). The resulting PPI data were exported and visualized using Cytoscape software (version 3.10.3). Network topology parameters, including degree centrality, betweenness centrality, and closeness centrality, were calculated using NetworkAnalyzer. Nodes within the top 20% according to degree centrality were regarded as hub targets for subsequent prioritization. Degree centrality was used as the primary criterion for ranking candidate docking targets, whereas betweenness and closeness centrality were used as supplementary criteria, particularly when nodes showed identical degree values.

### 4.5. GO and KEGG Functional Enrichment Analysis

Gene Ontology (GO) enrichment analysis (molecular function domain) and Kyoto Encyclopedia of Genes and Genomes (KEGG) pathway analysis were performed for the intersecting targets of UDCA and TUDCA using R (version 4.3.0) and the Bioconductor framework [36]. The R packages clusterProfiler, org.Hs.eg.db, colorspace, and enrichplot were utilized [37]. The parameters for enrichment analysis were set as follows: *p*-value cutoff = 0.05, p-adjustment method = “BH” (Benjamini–Hochberg), and q-value cutoff = 0.05. The reference gene universe was defined as all human protein-coding genes. The top 20 significantly enriched GO terms and KEGG pathways were visualized using dot plots and bar plots based on GeneRatio, gene count, and adjusted *p*-values.

### 4.6. Construction of Compound–Target–Pathway–Disease Integrated Network

To elucidate the shared and specific pharmacological mechanisms of UDCA and TUDCA in AD, an integrated “compound–target–pathway–disease” network was constructed. This network included the two bile acids, their respective AD-intersecting targets, significantly enriched KEGG pathways, and the disease node (AD). The network diagrams in this study were produced by Cytoscape. We use compounds, target genes, and pathways including UDCA and TUDCA as nodes in the network. The connections between nodes demonstrate the relationship between compounds, targets, pathways, and diseases. The visual characteristics used by the network include the color of the elements and the shape of the elements. This integrated network presents a comparative analysis of mechanistic convergence and divergence between UDCA and TUDCA in the context of Alzheimer’s disease.

### 4.7. Molecular Docking

Docking followed the same paradigm as our computational pipeline: receptors/ligands were prepared in PyMOL (Version 2.4.0a0) & Open Babel (Version 2.4.1). Protein and ligand preparation was carried out using AutoDock Tools, including the removal of water molecules, addition of hydrogen atoms, and assignment of Gasteiger charges. The prepared receptor and ligand were saved in PDBQT format. Docking simulations were conducted using AutoDock Vina (Version 1.2.3), with the docking grid defined to cover the active site of targets. AutoDock Vina generated nine poses per target and binding energy was the primary ranking metric. For each target, the lowest-energy pose (mode 1) was retained for interaction analysis (hydrogen bonds/salt bridges and hydrophobic contacts). Known inhibitors or activators were not included as reference ligands because the objective of this study was to compare the predicted interaction patterns of UDCA and TUDCA under a consistent docking framework rather than to evaluate competitive binding against established pharmacological modulators. Therefore, docking results were interpreted as structural hypotheses rather than evidence of target activation or inhibition. Docking grid boxes are summarized in Table 4 and Table 5 (center/size for each receptor–ligand pair). To ensure that receptor metadata reflect exactly the structures used in docking, we programmatically parsed the curated PDB files (chains, residual HETATM/ions, water, altLoc, occupancy) and compiled a QC table (Table 6).

Representative targets for molecular docking were prioritized primarily according to their topological importance in the corresponding PPI networks. Degree centrality was used as the principal ranking criterion, with betweenness and closeness centrality serving as supplementary measures when required to distinguish nodes with similar or identical degree values. For TUDCA, the six selected targets (BCL2, PTGS2, MTOR, JAK2, KIT, and PIK3CA) represented the six highest-ranked targets according to degree centrality. For UDCA, SRC, ESR1, EGFR, PPARA, and RXRA were among the five highest-ranked targets, whereas CYP19A1 shared the next-highest degree value with RARA and NR0B2 but showed higher betweenness and closeness centrality and was therefore prioritized. To complement the PPI-prioritized docking analysis and to enable ligand comparison within an identical receptor environment, GPBAR1 and MAPK14, which were present in both the UDCA–AD and TUDCA–AD target sets, were additionally examined by same-target docking. These two targets were selected from the shared-target structural screening because suitable ligand-bound receptor structures were available for docking validation and because they represented distinct shared target contexts relevant to the present analysis. For each shared target, UDCA and TUDCA were docked to the same receptor structure using the same binding-site definition, grid box, and docking parameters. Docking-score comparisons were restricted to the two ligands within each receptor system and were not used for cross-protein affinity comparisons. The availability and suitability of experimentally resolved protein structures were subsequently confirmed before molecular docking. Detailed topology parameters and target rankings are provided in Supplementary Tables S8 and S9.

Protein structures used for docking were retrieved from the Protein Data Bank (PDB) according to a predefined selection strategy. In order to better reflect the binding of bile acids to disease-related targets and to ensure consistency, we determined the following screening criteria. Priority was given to protein structures of human origin, structures that were experimentally resolved (X-ray resolved structures), PDB entries containing cocrystalline ligands, and structures with relatively complete functional domains, clear ligand binding regions, and very few missing residues around the docking pocket. When there were multiple PDB entries at the same target, a structure with better binding site integrity and overall suitable for small molecule docking was selected. Based on these criteria, the following receptor structures were used: CYP19A1 (3S79), EGFR (4WKQ), ESR1 (2IOG), PPARA (6KAX), RXRA (5MKU), SRC (1YOL), BCL2 (8HTS), JAK2 (3UGC), KIT (1T46), MTOR (4DRI), PIK3CA (7JIU), PTGS2 (3LN1), GPBAR1 (7CFN) and MAPK14 (4R3C).

### 4.8. Redocking Validation of the Molecular Docking Protocol

To evaluate the ability of the docking protocol to reproduce experimentally observed ligand-binding conformations, native-ligand redocking was performed for all 14 unique receptor structures used in the molecular docking analyses. For each receptor, the co-crystallized ligand was extracted from the corresponding PDB structure and subsequently redocked into its original binding site using the same receptor preparation procedure and docking protocol applied in the UDCA and TUDCA docking analyses. The redocked ligand pose was then compared with the corresponding crystallographic pose, and the root-mean-square deviation (RMSD) between the two conformations was calculated using PyMOL (Version 2.4.0a0) to evaluate pose reproduction. An RMSD value below 2.0 Å was used as the conventional criterion for satisfactory reproduction of the experimentally observed binding mode. RMSD values for all 14 receptor–native ligand systems are provided in Supplementary Table S7.

### 4.9. Molecular Dynamics Simulation

To further evaluate the stability and dynamic behavior of ligand–receptor complexes identified by comparative docking, molecular dynamics (MD) simulations were performed for the shared target GPBAR1 with UDCA and TUDCA. The GPBAR1 structure used for simulation was obtained from the Protein Data Bank (PDB ID: 7CFN), and the docking-derived ligand conformations were used as initial structures.

MD simulations were conducted using the AMBER molecular dynamics package. The protein was parameterized using the ff14SB force field, while ligand parameters were generated using the General Amber Force Field (GAFF2). Each complex was solvated in a TIP3P water box with a minimum distance of 10 Å from the solute, and Na+/Cl− ions were added to neutralize the system and maintain physiological ionic strength (0.15 M).

Prior to production simulation, energy minimization, gradual heating, and equilibration steps were performed under constant volume and pressure conditions. A 100 ns production simulation was subsequently conducted under constant temperature and pressure conditions. Trajectory analyses included root-mean-square deviation (RMSD), root-mean-square fluctuation (RMSF), radius of gyration (Rg), solvent accessible surface area (SASA), ligand RMSD, hydrogen-bond analysis, and MM/GBSA binding free energy calculations.

The dynamic stability and interaction characteristics of UDCA–GPBAR1 and TUDCA–GPBAR1 complexes were compared to investigate whether taurine conjugation influenced ligand accommodation and receptor interaction patterns.

### 4.10. In Silico ADMET and Physicochemical Property Prediction

To comparatively characterize the predicted physicochemical, drug-likeness, pharmacokinetic, and toxicity-related profiles of UDCA and TUDCA, in silico predictions were performed using the ADMETlab 3.0 platform (https://admetlab3.scbdd.com/; accessed on 11 April 2026). The chemical structures of UDCA (CAS No. 128-13-2) and TUDCA (CAS No. 14605-22-2) were obtained from PubChem and submitted to ADMETlab 3.0 in SMILES format. Predicted descriptors and endpoints included molecular weight, topological polar surface area (TPSA), lipophilicity- and solubility-related properties, Caco-2 permeability, human intestinal absorption (HIA), oral bioavailability-related predictions, plasma protein binding, unbound fraction, blood–brain barrier (BBB) permeability, cytochrome P450 (CYP)-related interactions, clearance, half-life, and selected toxicity-related endpoints [38,39]. Lipinski-related parameters were considered indicators of physicochemical properties and drug-likeness rather than direct evidence of in vivo pharmacokinetic behavior. Selected parameters were summarized in tables and radar plots to facilitate comparison of the in silico physicochemical and ADMET profiles of UDCA and TUDCA. Because this part of the results is derived from the computational prediction platform, it should be considered an in silico estimate rather than an in vivo experimental assay.

## 5. Conclusions

This study was designed to compare the predicted AD-related mechanisms of UDCA and TUDCA from the perspectives of target overlap, pathway preference, and representative ligand–target binding. The results showed that the two bile acids shared a considerable number of AD-related targets, indicating partial mechanistic convergence. At the same time, their dominant network features were clearly different: UDCA was more strongly linked to nuclear receptor-associated and metabolic regulatory pathways, whereas TUDCA showed greater relevance to survival signaling, anti-apoptotic processes, and neuroactive signaling pathways. These differences were further supported by molecular docking analyses, which provided structural context for distinct predicted interaction patterns between the two compounds. Furthermore, no combination-response or target-engagement experiments were performed for UDCA and TUDCA. The partially distinct or potentially complementary predicted target profiles identified in this study should not be interpreted as evidence of pharmacological synergy, additivity, or enhanced therapeutic efficacy of combined treatment.

Our findings indicate that UDCA and TUDCA should not be viewed as mechanistically identical in the context of AD. Instead, they appear to represent two partially overlapping but computationally distinct molecular profiles in the context of AD-associated mechanisms. These results provide a clearer comparative basis for future validation studies, but should be interpreted as mechanistic inference rather than direct evidence of therapeutic efficacy.

## Figures and Tables

**Figure 1:**
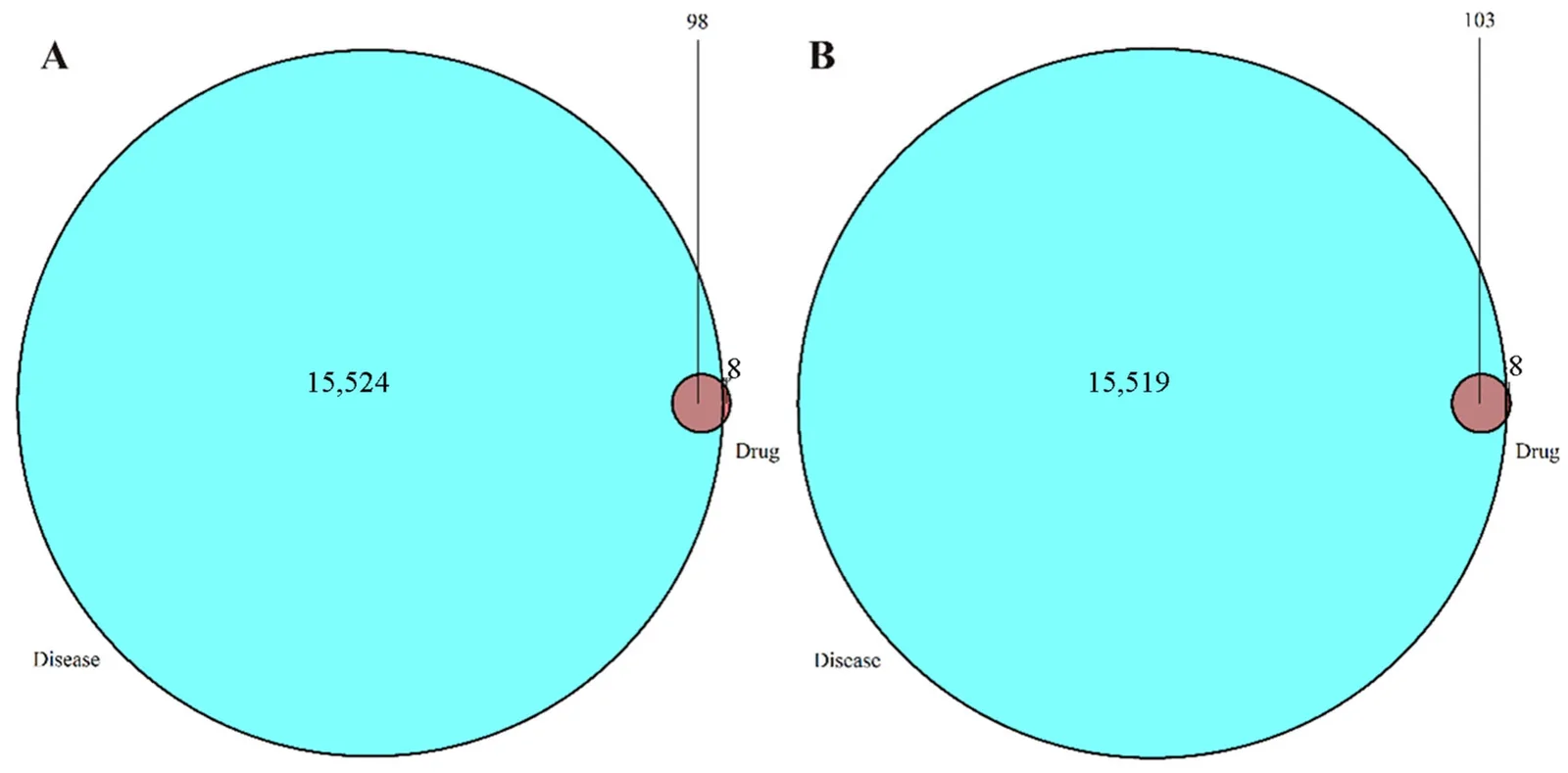
Venn diagrams of compound-predicted targets and Alzheimer’s disease (AD)-related genes. (**A**) Ursodeoxycholic acid (UDCA). (**B**) Tauroursodeoxycholic acid (TUDCA). AD-related genes were collected from GeneCards using the keyword “Alzheimer’s disease”. Genes with a relevance score > 1 were initially selected and combined with OMIM-derived genes after deduplication. Red circles indicate predicted compound targets, blue circles indicate AD-related genes, and overlapping regions represent intersection targets.

**Figure 2:**
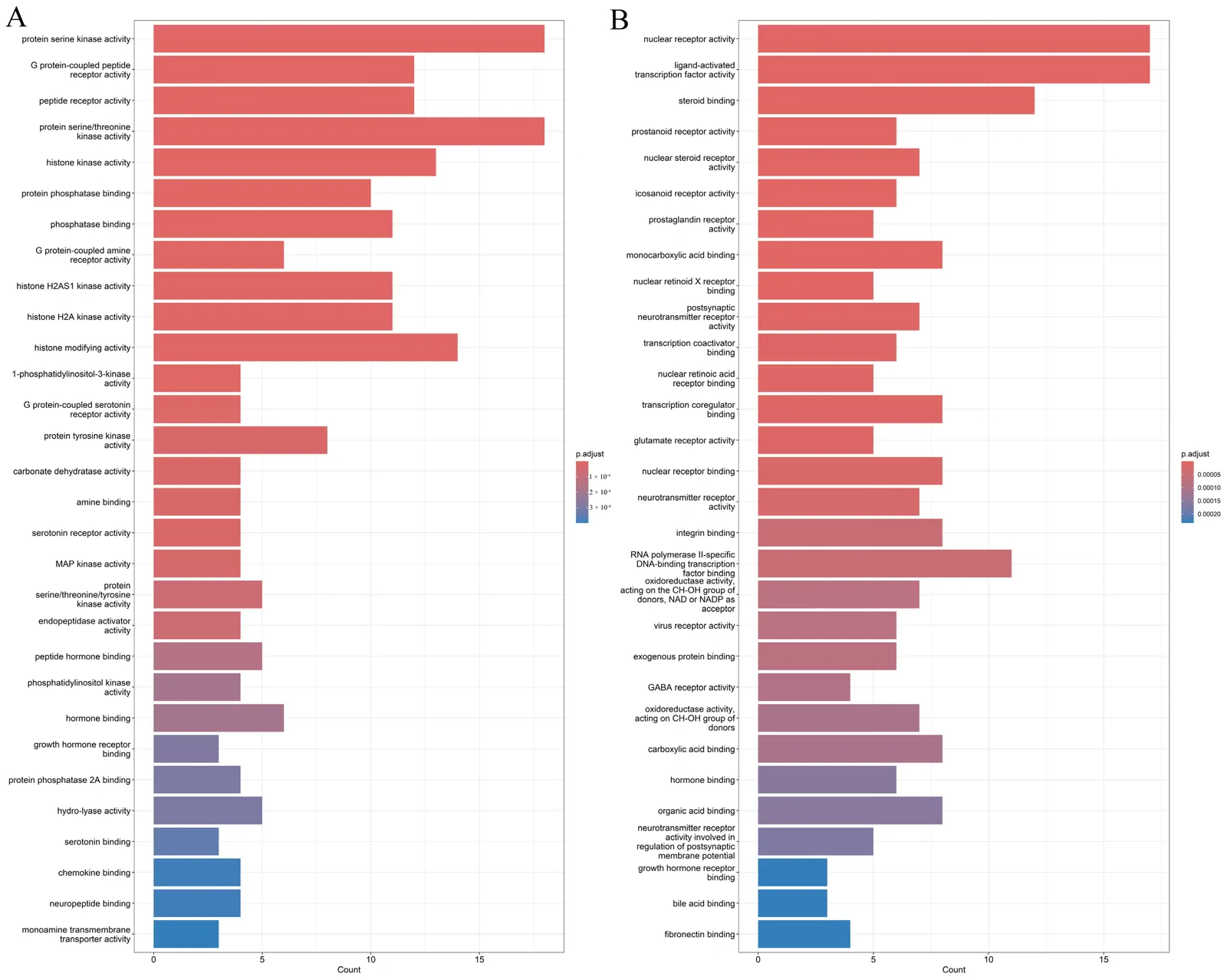
Analysis examining molecular function using the GO approach for target genes of UDCA and TUDCA that relate to Alzheimer’s disease. (**A**) Targets where TUDCA and Alzheimer’s disease intersect. (**B**) Targets where UDCA and Alzheimer’s disease intersect. The top enriched molecular function terms are shown based on clusterProfiler analysis using *p*-value cutoff = 0.05, Benjamini–Hochberg adjustment, and q-value cutoff = 0.05. The x-axis represents gene count, and color indicates adjusted *p*-value (p.adjust).

**Figure 3:**
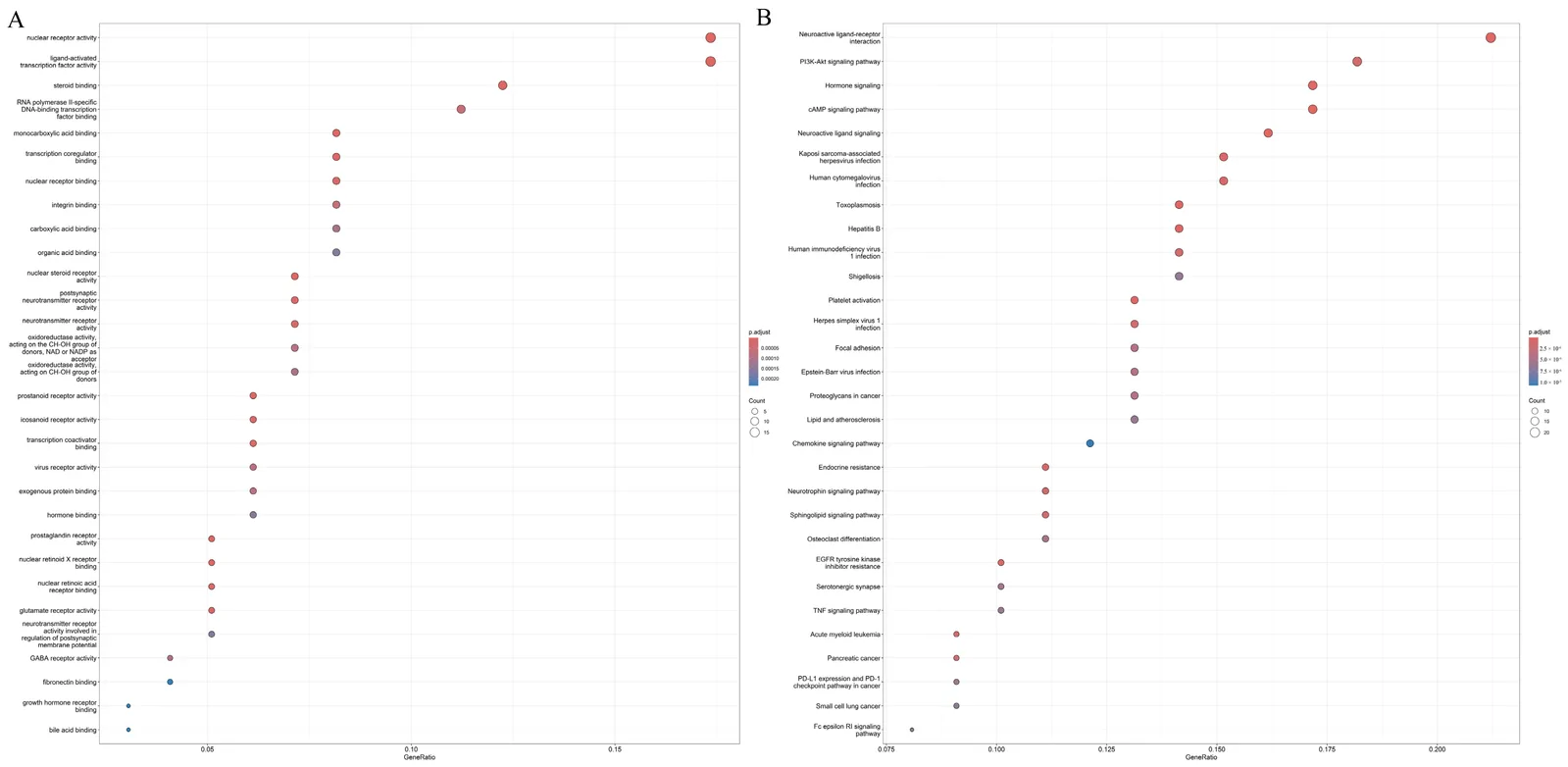
KEGG pathway enrichment analysis of UDCA and TUDCA intersection targets that associate with Alzheimer’s disease. (**A**) UDCA-AD intersection targets; (**B**) TUDCA-AD intersection targets. Significantly enriched pathways were identified using clusterProfiler with *p*-value cutoff = 0.05, Benjamini–Hochberg correction, and q-value cutoff = 0.05. The x-axis shows GeneRatio, dot size indicates gene count, and color represents adjusted *p*-value.

**Figure 4:**
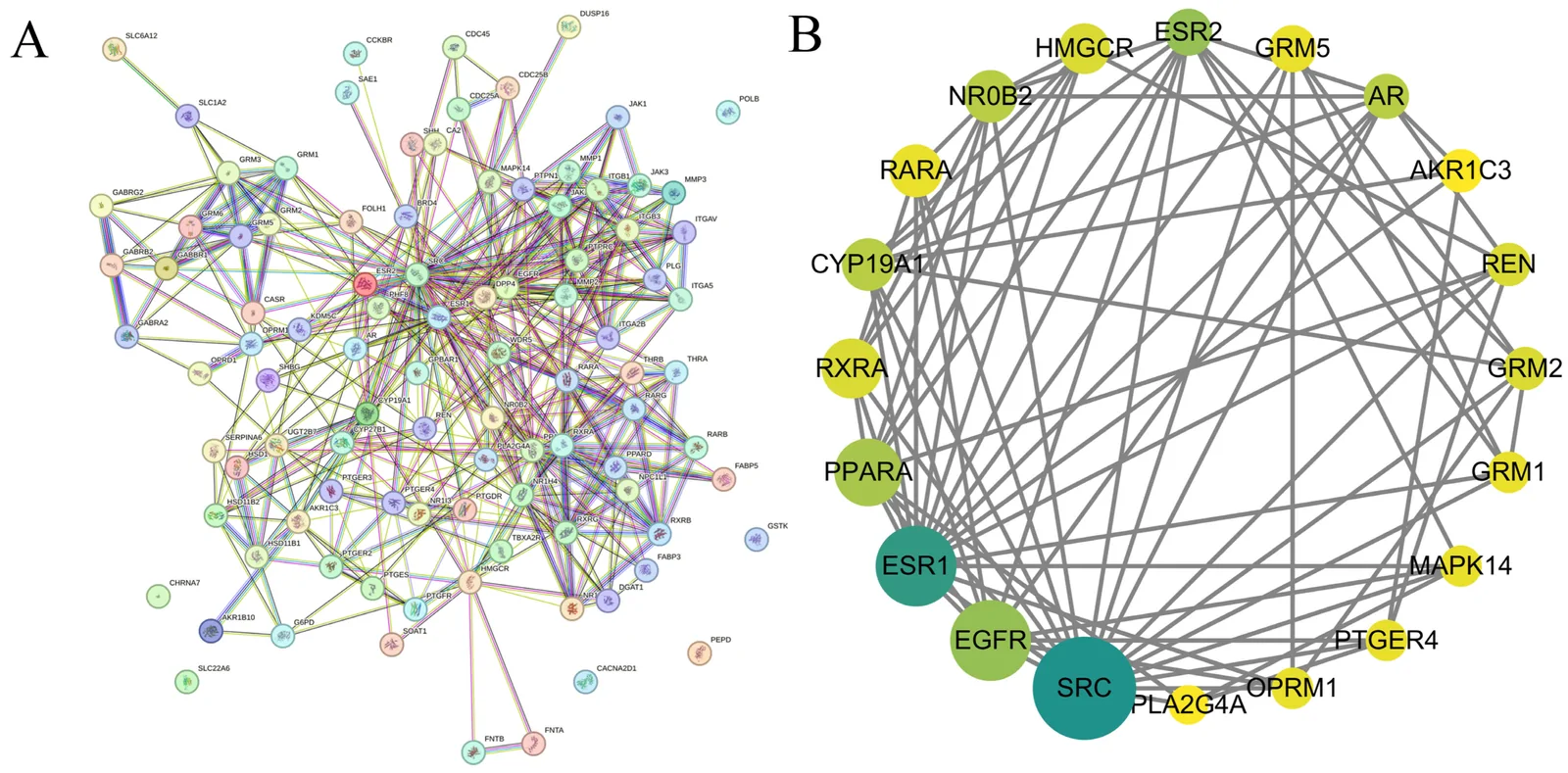
Protein–protein interaction (PPI) network of UDCA-AD intersection targets. (**A**) Full PPI network constructed in STRING (version 11.5) with organism restricted to Homo sapiens and a minimum interaction score of 0.7 (high confidence). (**B**) Hub-gene subnetwork visualized in Cytoscape (version 3.10.3); hub genes were defined as the top 20% of nodes ranked by degree. Node size represents degree centrality, and node color represents different levels of topological centrality.

**Figure 5:**
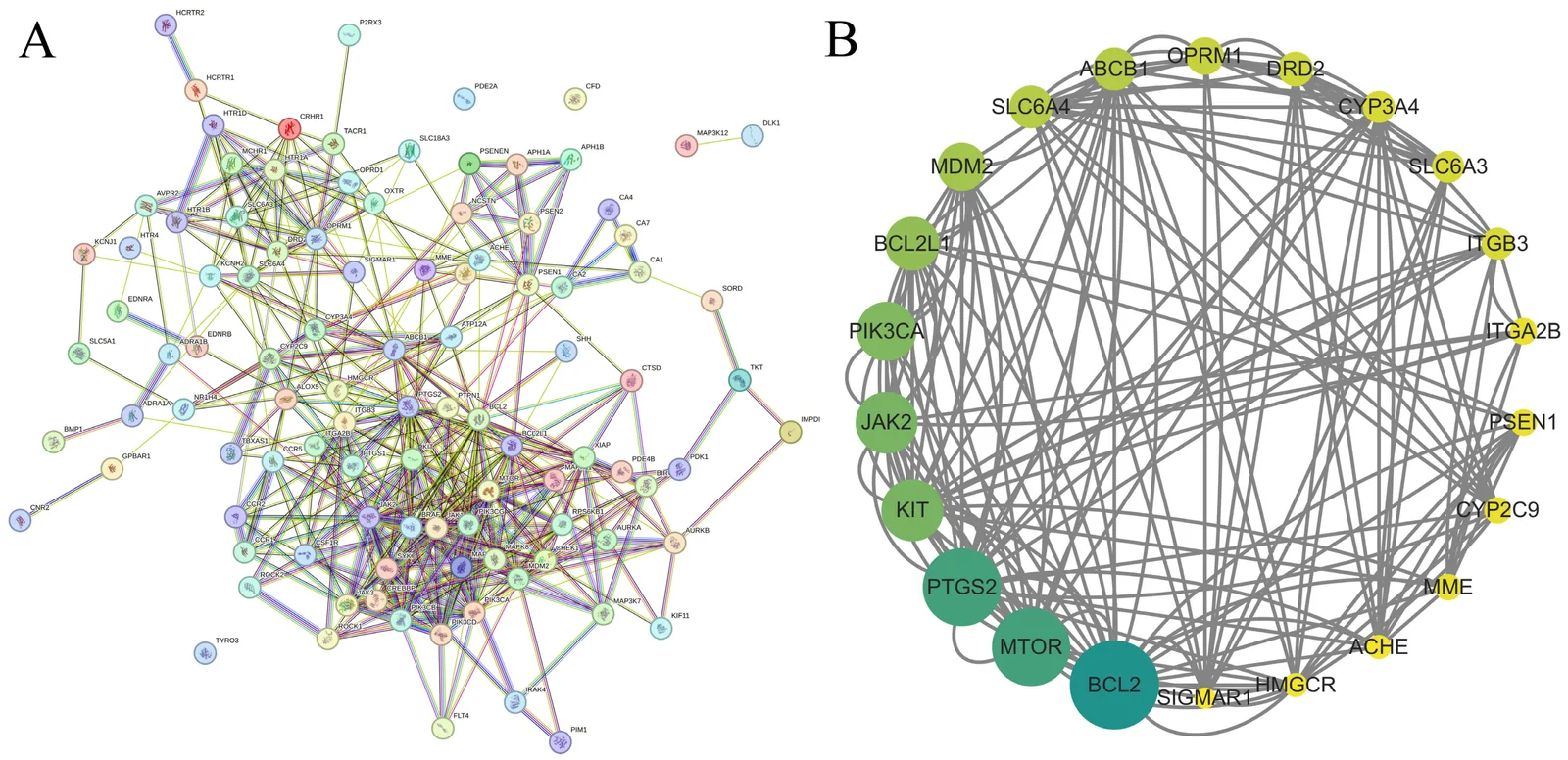
Protein–protein interaction (PPI) network of TUDCA-AD intersection targets. (**A**) Full PPI network constructed in STRING (version 11.5) with organism restricted to Homo sapiens and a minimum interaction score of 0.7 (high confidence). (**B**) Hub-gene subnetwork visualized in Cytoscape (version 3.10.3); hub genes were defined as the top 20% of nodes ranked by degree. Node size represents degree centrality, and node color represents different levels of topological centrality.

**Figure 6:**
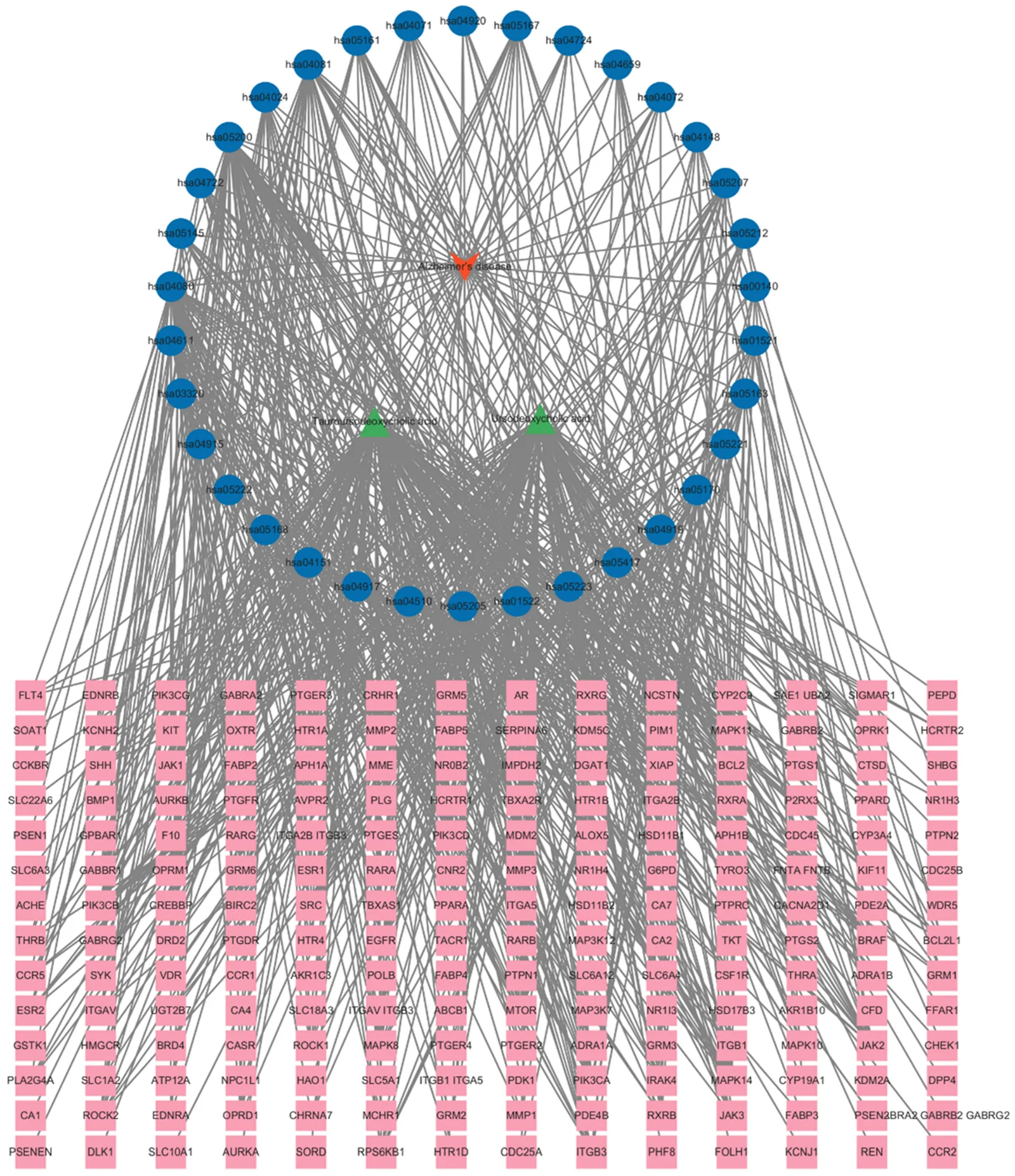
Integrated compound–target–pathway–disease network of UDCA and TUDCA in Alzheimer’s disease. The network was constructed in Cytoscape using the two compounds, their AD-intersection targets, significantly enriched KEGG pathways, and the disease node. Red node: AD; green nodes: compounds; pink nodes: target genes; blue nodes: enriched pathways. Edges represent compound–target, target–pathway, and target–disease associations.

**Figure 7:**
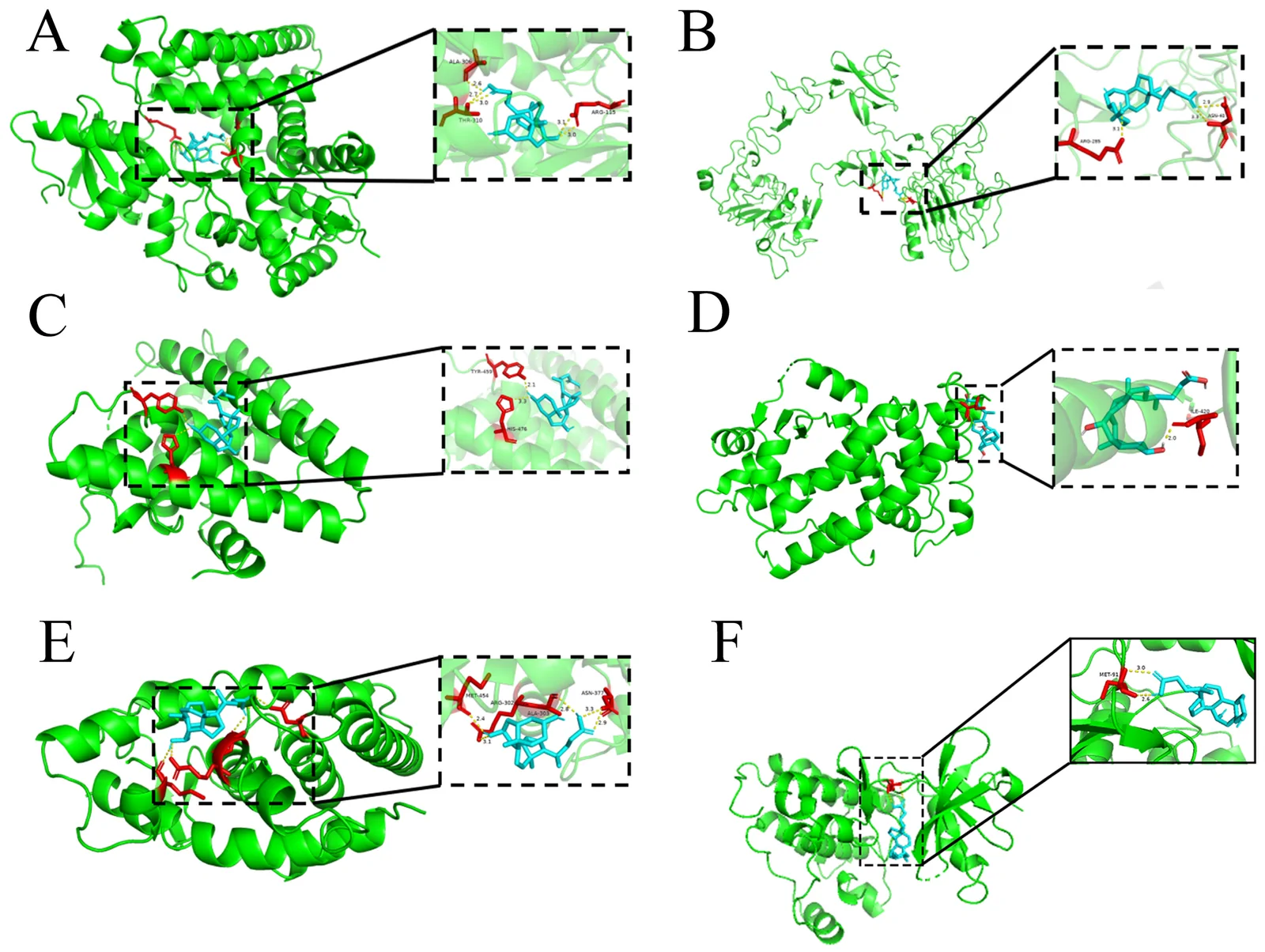
Representative docking poses of UDCA with six prioritized AD-related targets. Docking was performed using AutoDock Vina v1.2.3, and the lowest-energy pose (mode 1) was used for visualization. Receptors are shown in green, UDCA in cyan sticks, and key interacting residues in red. Panels correspond to (**A**) CYP19A1 (3S79), (**B**) EGFR (4WKQ), (**C**) ESR1 (2IOG), (**D**) PPARA (6KAX), (**E**) RXRA (5MKU), and (**F**) SRC (1YOL).

**Figure 8:**
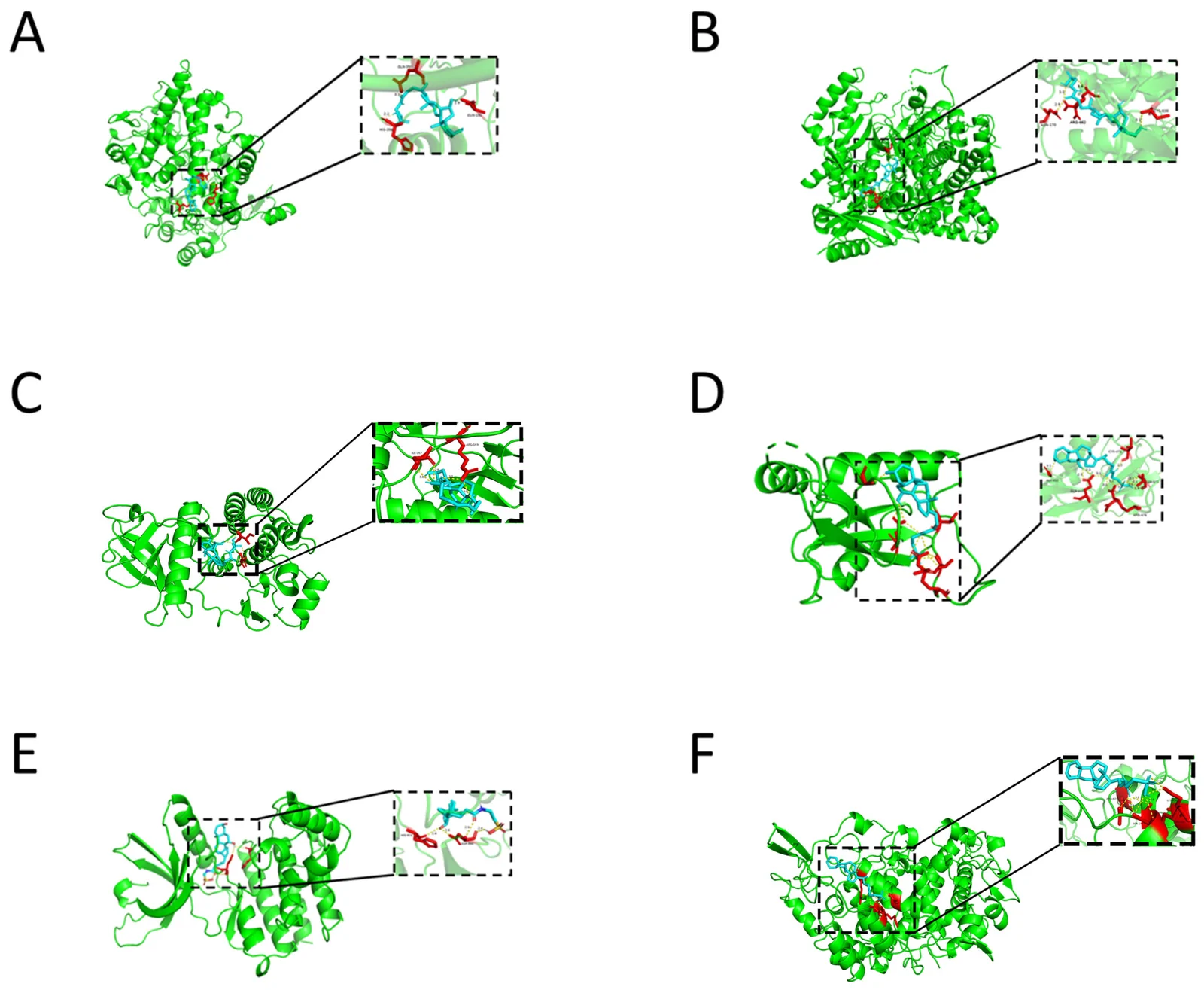
Representative docking poses of TUDCA with six prioritized AD-related targets. Docking was performed using AutoDock Vina v1.2.3, and the lowest-energy pose (mode 1) was used for visualization. Receptors are shown in green, TUDCA in cyan, and key interacting residues in red. Panels correspond to (**A**) PIK3CA (7JIU), (**B**) JAK2 (3UGC), (**C**) KIT (1T46), (**D**) MTOR (4DRI), (**E**) BCL2 (8HTS), and (**F**) PTGS2 (3LN1).

**Figure 9:**
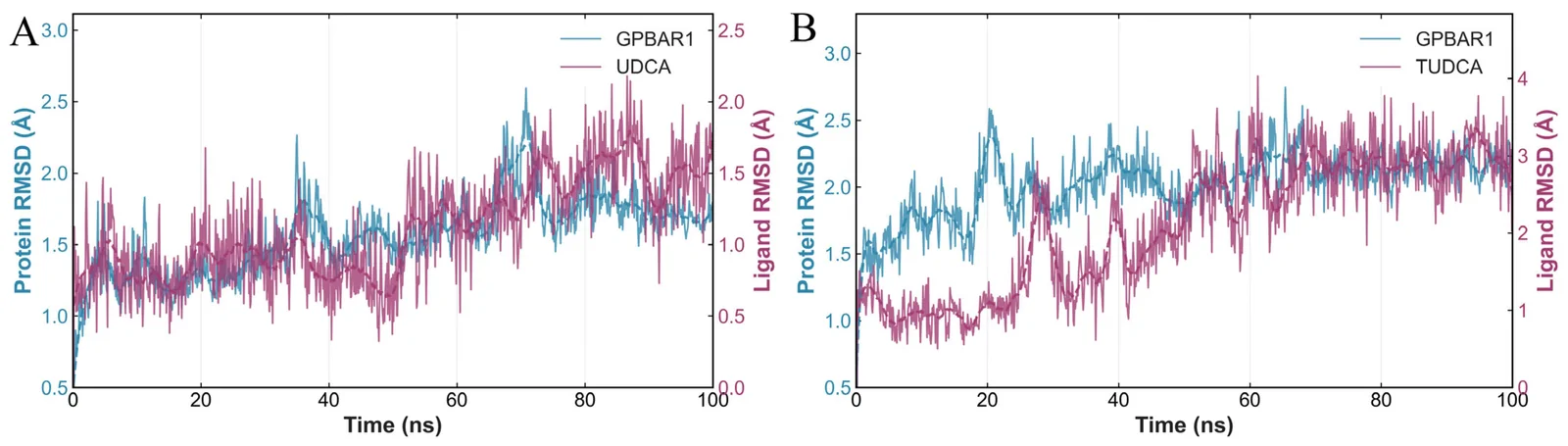
Structural stability analysis of GPBAR1–UDCA and GPBAR1–TUDCA complexes during 100 ns molecular dynamics simulations. (**A**) Root mean square deviation (RMSD) profiles of the GPBAR1–UDCA complex for protein backbone and ligand atoms. (**B**) RMSD profiles of the GPBAR1–TUDCA complex for protein backbone and ligand atoms. The RMSD trajectories indicate that both complexes reached equilibrium during the simulation period, supporting the structural stability of ligand-bound GPBAR1 conformations.

**Figure 10:**
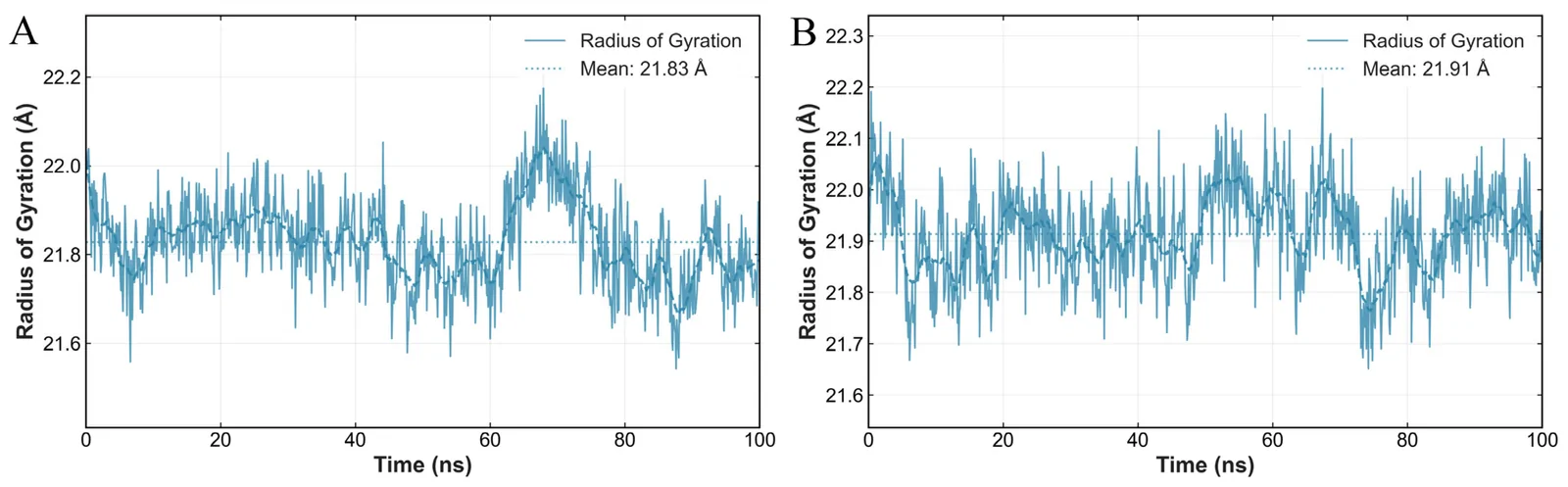
Radius of gyration (Rg) during 100 ns molecular dynamics simulations of GPBAR1–UDCA and GPBAR1–TUDCA complexes. (**A**) Rg analysis of the GPBAR1–UDCA complex. (**B**) Rg analysis of the GPBAR1–TUDCA complex.

**Figure 11:**
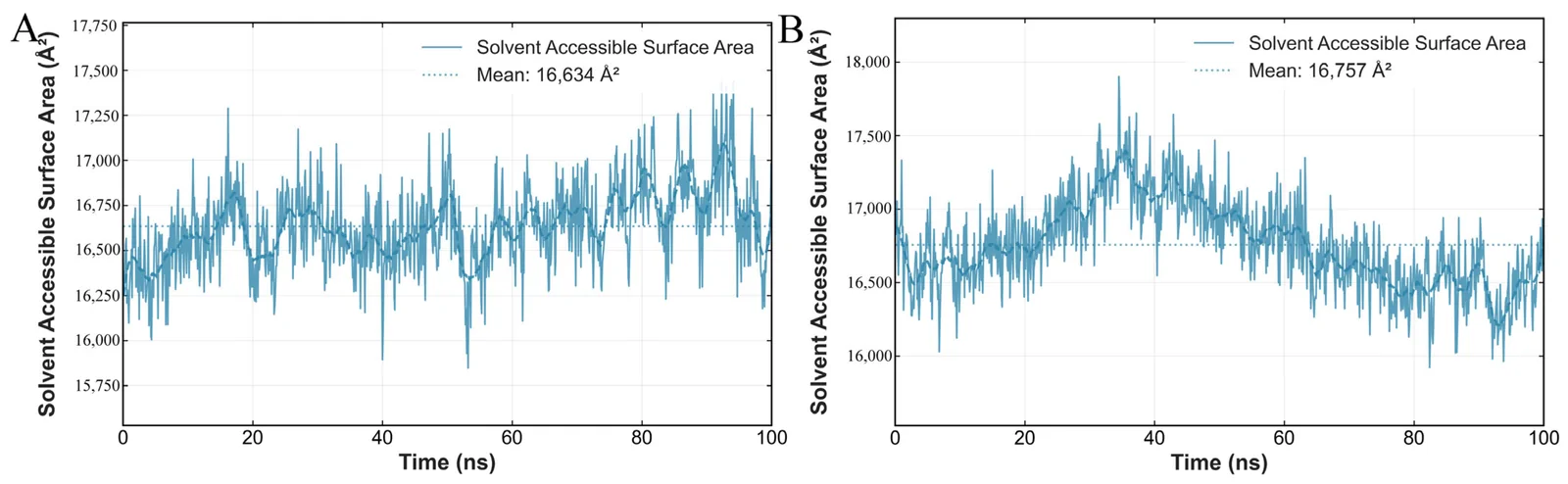
Solvent accessible surface area (SASA) analysis of GPBAR1–UDCA and GPBAR1–TUDCA complexes during 100 ns molecular dynamics simulations. (**A**) SASA profile of the GPBAR1–UDCA complex. (**B**) SASA profile of the GPBAR1–TUDCA complex. SASA values were calculated throughout the simulation trajectories to evaluate changes in solvent exposure and global conformational characteristics of GPBAR1 upon interaction with UDCA and TUDCA.

**Figure 12:**
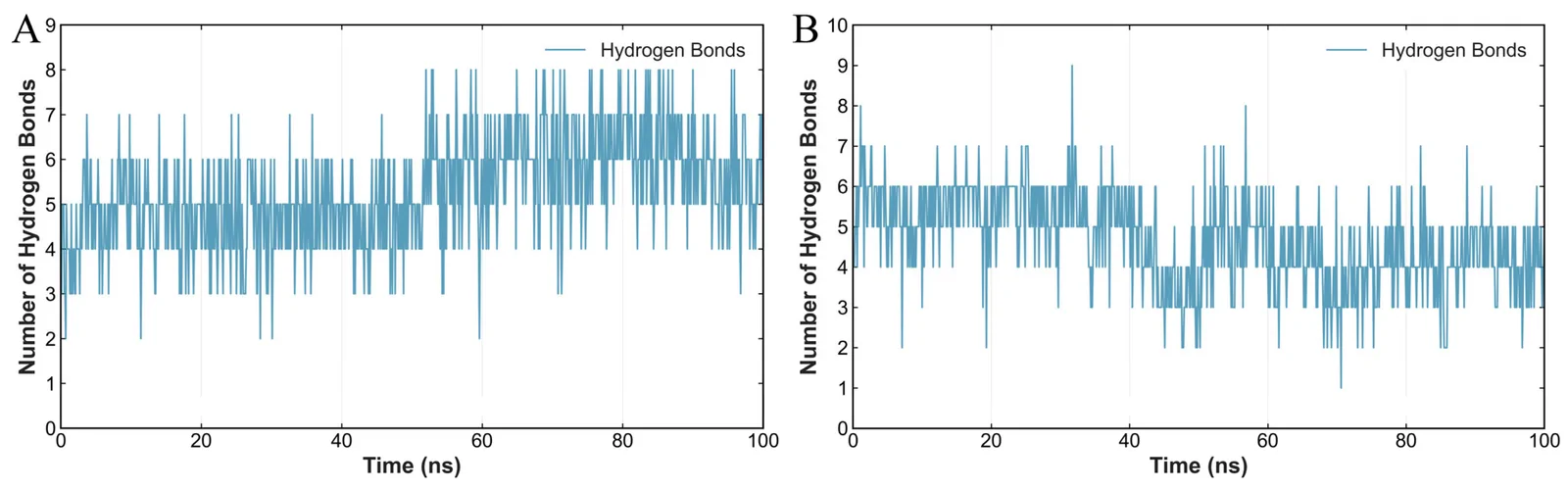
Dynamic hydrogen bond interactions between GPBAR1 and bile acids during molecular dynamics simulations. (**A**) Number of hydrogen bonds formed between GPBAR1 and UDCA throughout the 100 ns simulation. (**B**) Number of hydrogen bonds formed between GPBAR1 and TUDCA throughout the 100 ns simulation.

**Figure 13:**
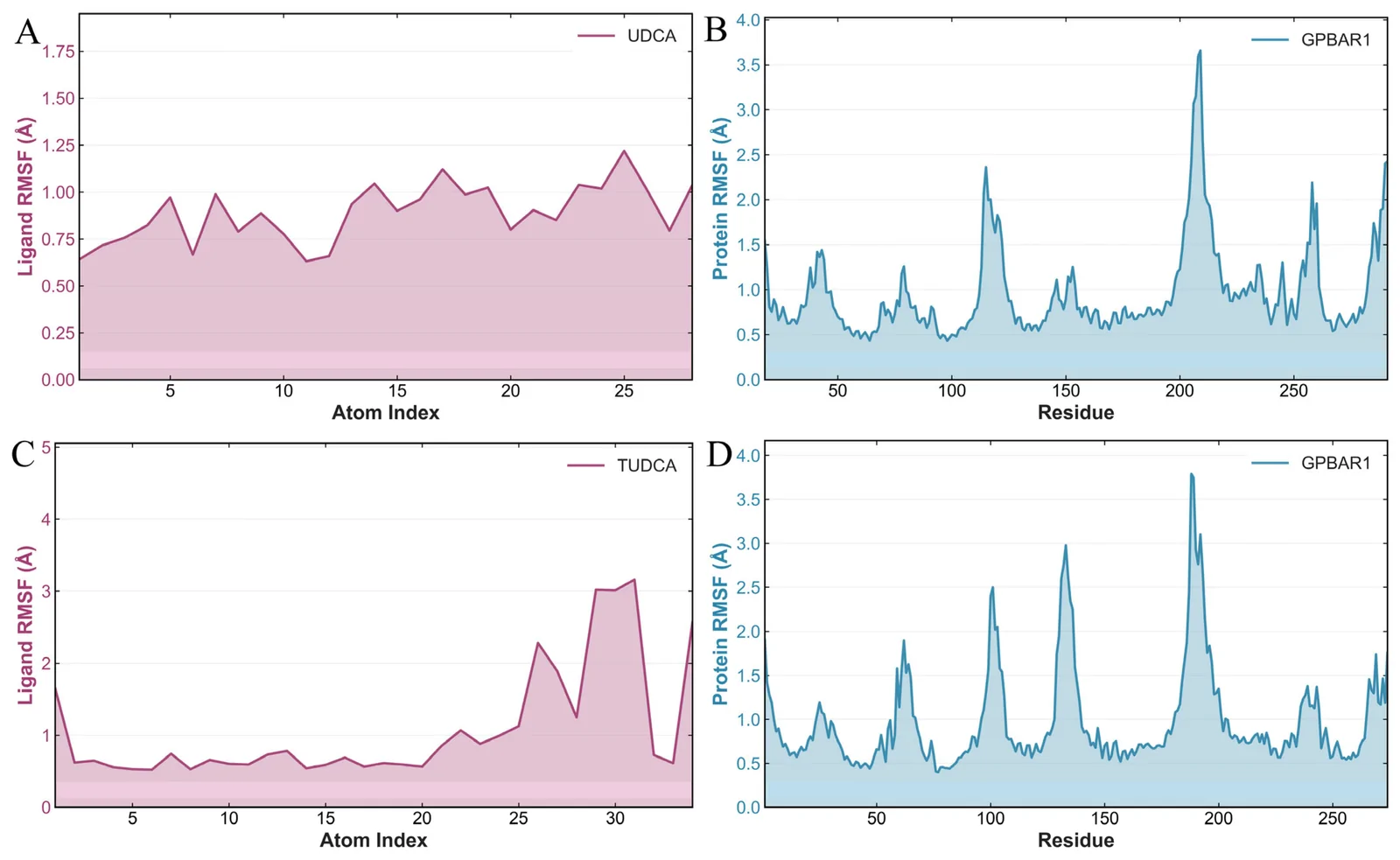
Root mean square fluctuation (RMSF) analysis of GPBAR1 residues and bound bile acids. (**A**,**B**) RMSF profiles of UDCA ligand atoms and GPBAR1 residues in the GPBAR1–UDCA complex. (**C**,**D**) RMSF profiles of TUDCA ligand atoms and GPBAR1 residues in the GPBAR1–TUDCA complex. RMSF values were calculated to characterize local flexibility changes during the 100 ns MD simulations.

**Figure 14:**
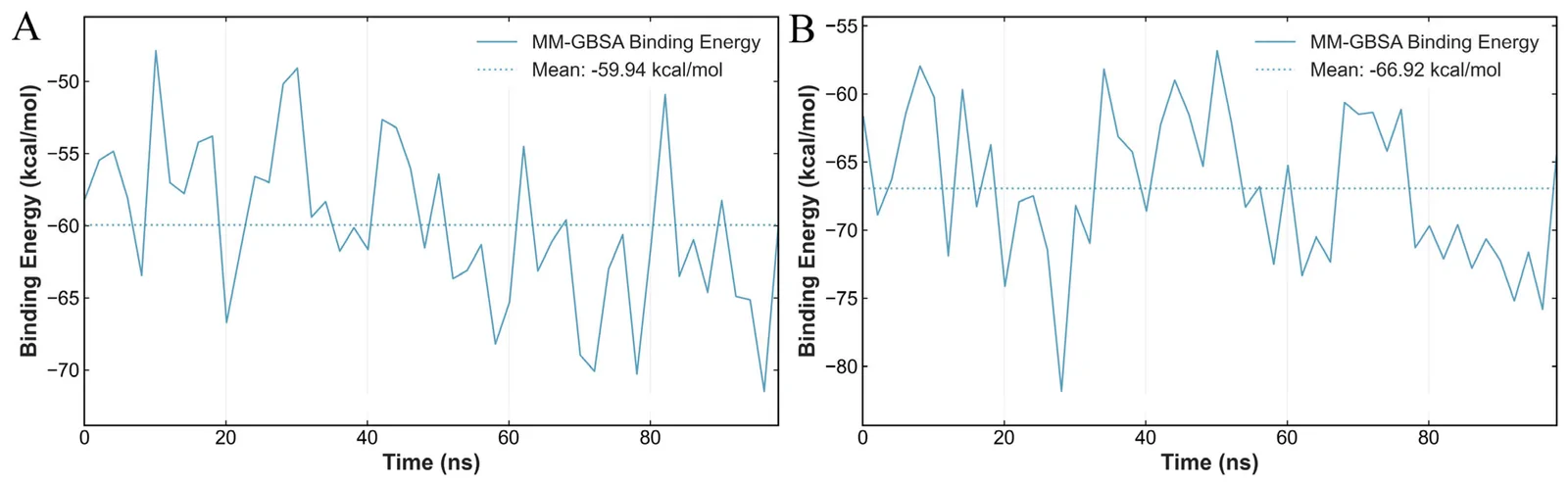
MM/GBSA binding free energy analysis of GPBAR1–UDCA and GPBAR1–TUDCA complexes. (**A**) Binding free energy trajectory of the GPBAR1–UDCA complex with an average binding energy of −59.94 kcal/mol. (**B**) Binding free energy trajectory of the GPBAR1–TUDCA complex with an average binding energy of −66.92 kcal/mol. The MM/GBSA results indicate a more favorable calculated binding free energy for TUDCA–GPBAR1 compared with UDCA–GPBAR1 under the simulated conditions.

**Figure 15:**
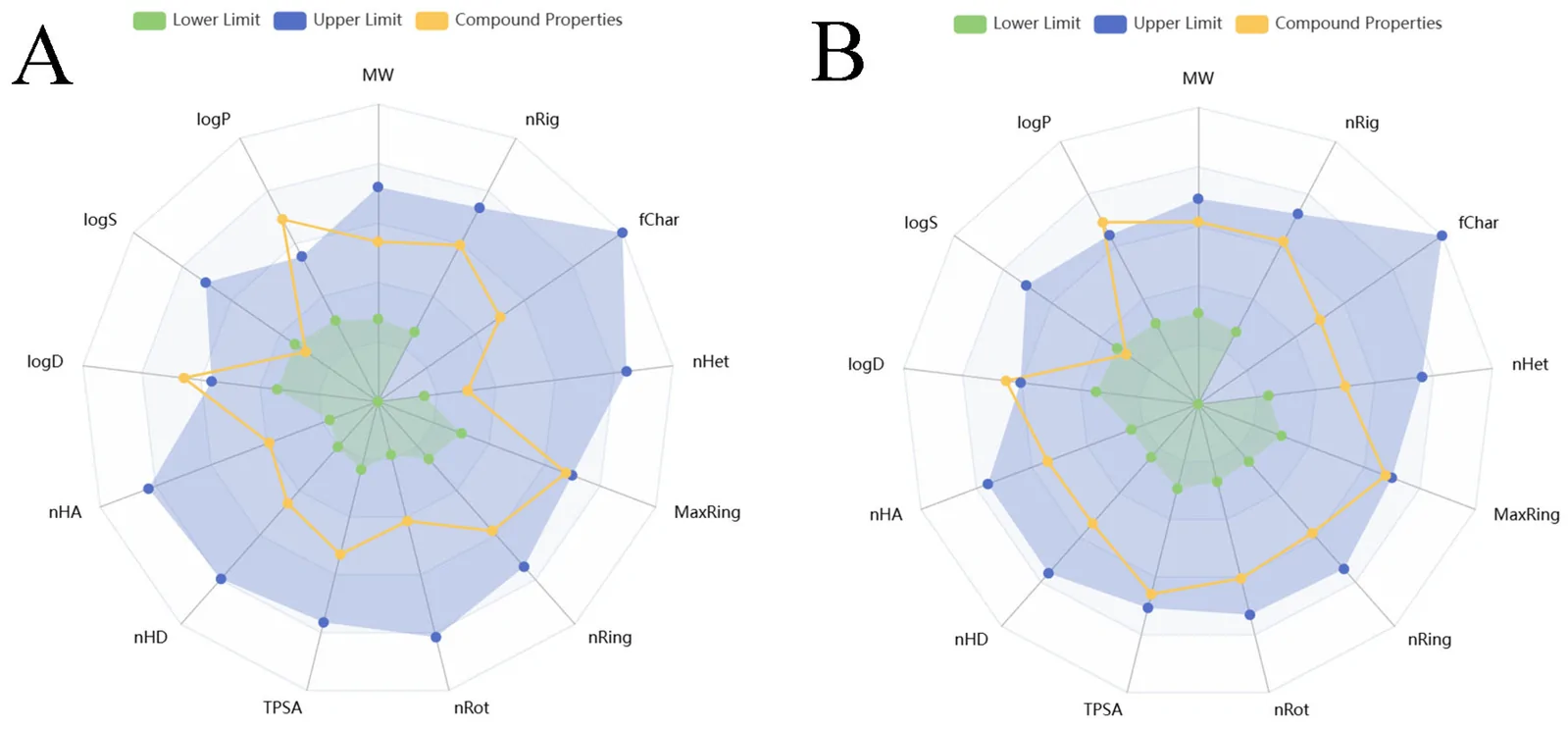
Radar plot comparison of physicochemical property profiles of UDCA and TUDCA. (**A**) UDCA. (**B**) TUDCA. The green area represents the lower recommended limit, the blue area indicates the upper recommended limit, and the yellow polygon corresponds to the calculated molecular properties of each compound. Parameters include molecular weight (MW), number of rigid bonds (nRig), formal charge (fChar), number of heteroatoms (nHet), maximum ring size (MaxRing), number of rings (nRing), number of rotatable bonds (nRot), topological polar surface area (TPSA), number of hydrogen bond donors (nHD), number of hydrogen bond acceptors (nHA), distribution coefficient (logD), aqueous solubility (logS), and partition coefficient (logP).

**Figure 16:**
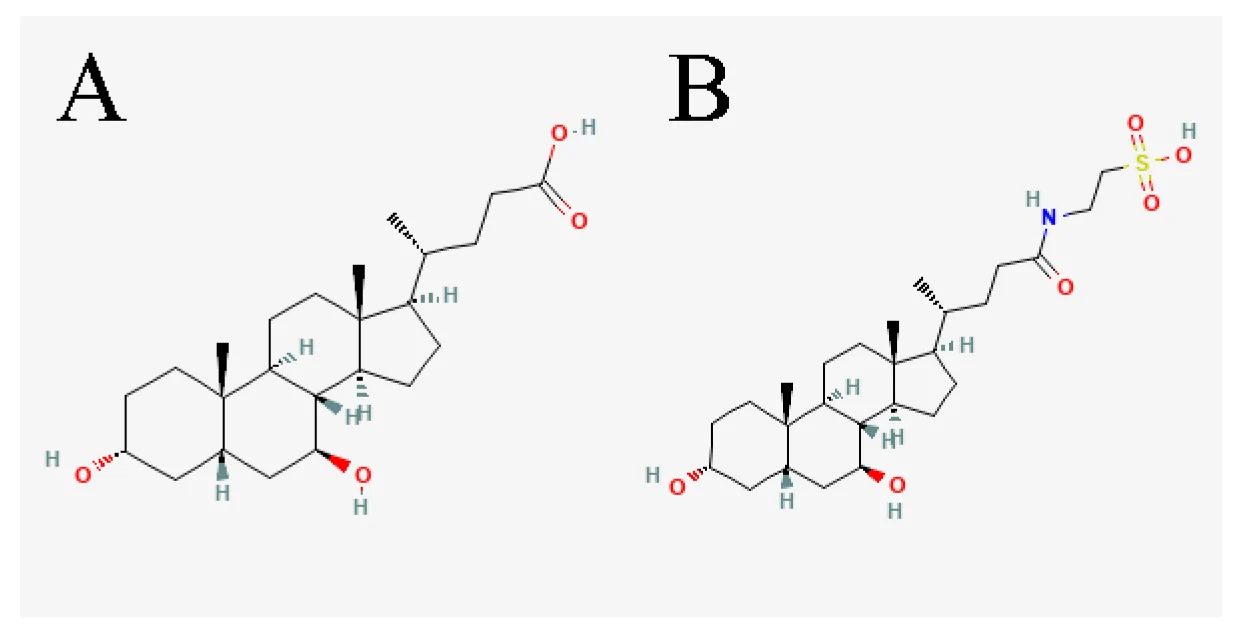
Chemical structures of the two bile acids analyzed in this study. (**A**) Ursodeoxycholic acid (UDCA). (**B**) Tauroursodeoxycholic acid (TUDCA). TUDCA is the taurine-conjugated derivative of UDCA, characterized by the presence of a taurine side chain linked to the terminal carboxyl group of the bile acid scaffold.

**Table 1:** Molecular docking results of UDCA with prioritized AD-related targets.

Target Protein (PDB ID)	Grid Center (x, y, z)	Grid Size (Å^3^)
CYP19A1 (3S79)	(83.347, 52.488, 46.452)	72 × 82 × 60
EGFR (4WKQ)	(7.547, 202.828, 24.235)	68 × 50 × 62
ESR1 (2IOG)	(23.694, 5.496, 20.525)	56.8 × 59.9 × 61.0
PPARA (6KAX)	(5.297, 1.981, 1.715)	58 × 50 × 66
RXRA (5MKU)	(21.644, −12.055, −11.285)	46 × 54 × 52
SRC (1YOL)	(9.958, 30.996, 25.681)	52 × 46 × 62

Predicted binding energies (kcal/mol) and representative interacting residues for CYP19A1, EGFR, ESR1, PPARA, RXRA, and SRC.

**Table 2:** Molecular docking results of TUDCA with prioritized AD-related targets.

Target Protein (PDB ID)	Grid Center (x, y, z)	Grid Size (Å^3^)
BCL2 (8HTS)	(9.601, 10.761, 12.200)	58 × 52 × 46
JAK2 (3UGC)	(18.804, −12.789, −15.463)	54 × 70 × 64
KIT (1T46)	(31.249, 24.459, 35.096)	58 × 60 × 58
MTOR (4DRI)	(35.041, 36.688, 42.573)	48 × 48 × 40
PIK3CA (7JIU)	(−3.436, 8.547, 18.758)	88 × 104 × 98
PTGS2 (3LN1)	(34.705, −27.124, −8.642)	62 × 82 × 72

Predicted binding energies (kcal/mol) and representative interacting residues for BCL2, JAK2, KIT, MTOR, PIK3CA, and PTGS2.

**Table 3:** Comparative molecular docking results of UDCA and TUDCA against shared AD-associated targets.

Target	Ligand	Binding Energy (kcal/mol)	Representative Interaction Residues
GPBAR1	UDCA	−9.4	Tyr89
GPBAR1	TUDCA	−9.1	Tyr89, Ser156, Ser157, Tyr240, Ser247
MAPK14	UDCA	−9.3	Ala74
MAPK14	TUDCA	−8.7	Lys76, Glu94, Asp191, Met132

QC parameters include atom/residue counts, HETATM/HOH counts, alternate locations, partial occupancies, and heterogroups.

**Table 4:** Docking grid box settings for UDCA targets.

Target Protein (PDB ID)	Binding Energy (kcal/mol)	Key Amino Acid Residues	Main Interaction Types
CYP19A1 (3s79)	−7.9	Ala306, Thr310, Arg115	Hydrogen Bond
EGFR (1yy9)	−7.0	Ile420, Arg207	Hydrogen Bond, Hydrophobic
ESR1 (2iog)	−7.1	Tyr459, His476	Hydrogen Bond
PPARA (6kax)	−8.1	Ile420	Hydrogen Bond
RXRA (5mku)	−7.0	Met454, Arg302, Ala303, Asn377	Hydrogen Bond
SRC (8hts)	−7.0	Met91	Hydrogen Bond, π-π stacking

Target protein (PDB ID), grid box center (x, y, z, Å), and grid box size (Å^3^) used for UDCA docking. Coordinates and sizes follow the docking configuration derived for CYP19A1 (3S79), EGFR (4WKQ), ESR1 (2IOG), PPARA (6KAX), RXRA (5MKU), and SRC (1YOL).

**Table 5:** Docking grid box settings for TUDCA targets.

Target Protein (PDB ID)	Binding Energy (kcal/mol)	Key Amino Acid Residues	Main Interaction Types
BCL2 (8hts)	−7.4	Tyr180, Arg183, His184	Hydrogen Bond, Hydrophobic
JAK2 (3ugc)	−9.3	His974, Asp994	Hydrogen Bond, Salt Bridge
KIT (2vif)	−8.5	Ile167, Arg169	Hydrogen Bond, Salt Bridge
MTOR (4dri)	−7.7	Ser2035	Hydrogen Bond
PIK3CA (7jiu)	−10.3	Asn170, Arg662, Cys838	Hydrogen Bond, Salt Bridge
PTGS2 (5f19)	−7.1	Arg425, Ser143, Lys418, Lys144	Hydrogen Bond

Target protein (PDB ID), grid box center (x, y, z, Å), and grid box size (Å^3^) used for TUDCA docking. Parameters are shown for BCL2 (8HTS), JAK2 (3UGC), KIT (1T46), MTOR (4DRI), PIK3CA (7JIU), and PTGS2 (3LN1).

**Table 6:** QC metrics for receptor structures used in UDCA and TUDCA docking.

Ligand	File	Pdb_id_Guess	Chains	Num_Chains	Num_Atoms	Num_Residues	Num_HETATM	Num_HOH	Nonwater_Het_Resnames	Ion_Resnames	Has_Altloc	Has_Partial_Occupancy	Remarks	Preference
TUDCA	PTGS2-3ln1_for_docking.pdb	3LN1	B	1	4474	551	0	0			TRUE	TRUE		2
	PIK3CA-7jiu_for_docking.pdb	7jiu	A	1	7409	900	0	0			TRUE	TRUE		2
	MTOR-4dri.pdb	4dri	B	1	837	96	0	0			TRUE	TRUE		0
	KIT-1t46_for_docking.pdb	1T46	A	1	1095	131	4	0	EDO		TRUE	TRUE		2
	JAK2_3ugc_for_docking.pdb		A	1	2303	274	0	0			TRUE	TRUE		2
	CYP19A1_3s79 no small molecule and water.pdb	3S79	A	1	3668	452	0	0			TRUE	TRUE		1
UDCA	EGFR_4wkq.pdb	4WKQ	A	1	4653	613	0	0			FALSE	FALSE		0
	CYP19A1_3s79 no small molecule and water.pdb	3S79	A	1	3668	452	0	0			TRUE	TRUE		1
	SRC 8hts no small molecule and water.pdb	8HTS	A	1	1285	150	0	0			TRUE	TRUE		1
	PPARA 6kax.pdb	6KAX	A	1	2455	265	221	158	GOL, PLM		TRUE	TRUE		0
	RXRA_5mku.pdb	5MKU	A	1	1756	208	0	0			TRUE	TRUE		0
	ESR1 2iog no small molecule and water.pdb	2IOG	A	1	1866	234	0	0			FALSE	FALSE		1

## Data Availability

The datasets analyzed during the current study are publicly available from GeneCards (https://www.genecards.org; accessed on 24 May 2025), OMIM (https://www.omim.org; accessed on 24 May 2025), SwissTargetPrediction (http://www.swisstargetprediction.ch; accessed on 24 May 2025), and STRING (https://string-db.org; accessed on 24 May 2025). All data supporting the findings of this study are included in the article or are available from the corresponding author upon reasonable request.

## Supplementary Materials

The following supporting information can be downloaded at: https://www.mdpi.com/article/10.3390/ph19101512/s1, Table S1: GO molecular function enrichment results for UDCA-AD intersection targets; Table S2: GO molecular function enrichment results for TUDCA-AD intersection targets; Table S3: KEGG pathway enrichment results for TUDCA-AD intersection targets; Table S4: KEGG pathway enrichment results for UDCA-AD intersection targets; Table S5: UDCA vs. TUDCA; Table S6: Redocking RMSD values; Table S7: Network topology analysis of UDCA-related targets and selection of candidate targets for molecular docking; Table S8: Network topology analysis of TUDCA-related targets and selection of candidate targets for molecular docking; Table S9: Effect of different GeneCards relevance score thresholds on AD-associated targets and drug-disease intersections; Table S10: Stability of UDCA-associated hub targets under different GeneCards relevance score thresholds; Table S11: Stability of TUDCA-associated hub targets under different GeneCards relevance score thresholds; Table S12: Summary of independent target support; Table S13: Independent evidence supporting UDCA-associated key targets; Table S14: Predicted targets of ursodeoxycholic acid (UDCA) based on ChEMBL database analysis; Table S15: Predicted targets of tauroursodeoxycholic acid (TUDCA) based on ChEMBL database analysis.

## Abbreviations

The following abbreviations are used in this manuscript:

Abbreviation	Full Term
AD	Alzheimer’s disease
UDCA	Ursodeoxycholic acid
TUDCA	Tauroursodeoxycholic acid
PPI	Protein–Protein Interaction
GO	Gene Ontology
KEGG	Kyoto Encyclopedia of Genes and Genomes
PDB	Protein Data Bank
STRING	Search Tool for the Retrieval of Interacting Genes/Proteins
OMIM	Online Mendelian Inheritance in Man
CSF	Cerebrospinal fluid
APOE	Apolipoprotein E
PSEN1	Presenilin-1
NF-κB	Nuclear factor kappa-light-chain-enhancer of activated B cells
AMPK	AMP-activated protein kinase
MAPK	Mitogen-activated protein kinase
ROS	Reactive oxygen species
AR	Androgen receptor
RARA	Retinoic acid receptor alpha
RXRA	Retinoid X receptor alpha
ESR1	Estrogen receptor alpha
NR0B2 (SHP)	Nuclear receptor subfamily 0, group B, member 2
GRM1/2/5	Metabotropic glutamate receptors 1/2/5
OPRM1	Mu-opioid receptor 1
CHRNA7	Nicotinic acetylcholine receptor α7 subunit
JAK2	Janus kinase 2
MTOR	Mammalian target of rapamycin
PIK3CA	Phosphatidylinositol-4,5-bisphosphate 3-kinase catalytic subunit alpha
BCL2	B-cell lymphoma 2
PTGS2 (COX-2)	Prostaglandin-endoperoxide synthase 2
EGFR	Epidermal growth factor receptor
SRC	Proto-oncogene tyrosine-protein kinase Src
CYP19A1	Aromatase
DRD2	Dopamine D2 receptor
SLC6A4	Serotonin transporter
ACHE	Acetylcholinesterase
ADMET	Absorption, Distribution, Metabolism, Excretion and Toxicity
