# Supplementary material for: Comparative Network Pharmacology and Molecular Docking Analysis of Ursodeoxycholic Acid and Tauroursodeoxycholic Acid in Alzheimer’s Disease-Associated Molecular Networks
Source: Pharmaceuticals (Basel). 2026 Sep 23;19(10):1512. doi: 10.3390/ph19101512 (PMC13708223; doi:10.3390/ph19101512)
Supplement: Supplementary file 1 [file pharmaceuticals-19-01512-s001.zip › Supplementary Table S3.pdf]

KEGG pathway enrichment results for TUDCA-AD intersection targets

Rank	KEGG ID	Pathway	Count	GeneRatio	Adjusted p value
1	hsa05145	Toxoplasmosis	14	14/99	3.97×10^{-9}
2	hsa04081	Hormone signaling	17	17/99	2.22×10^{-8}
3	hsa04024	cAMP signaling pathway	17	17/99	2.22×10^{-8}
4	hsa04080	Neuroactive ligand-receptor interaction	21	21/99	2.22×10^{-8}
5	hsa04082	Neuroactive ligand signaling	16	16/99	2.22×10^{-8}
6	hsa04611	Platelet activation	13	13/99	4.20×10^{-8}
7	hsa05161	Hepatitis B	14	14/99	8.98×10^{-8}
8	hsa05167	Kaposi sarcoma-associated herpesvirus infection	15	15/99	1.01×10^{-7}
9	hsa01522	Endocrine resistance	11	11/99	2.64×10^{-7}
10	hsa01521	EGFR tyrosine kinase inhibitor resistance	10	10/99	3.64×10^{-7}
11	hsa05163	Human cytomegalovirus infection	15	15/99	5.10×10^{-7}
12	hsa05221	Acute myeloid leukemia	9	9/99	9.88×10^{-7}
13	hsa04151	PI3K-Akt signaling pathway	18	18/99	1.29×10^{-6}
14	hsa04722	Neurotrophin signaling pathway	11	11/99	1.29×10^{-6}
15	hsa05170	Human immunodeficiency virus 1 infection	14	14/99	1.29×10^{-6}
16	hsa04071	Sphingolipid signaling pathway	11	11/99	1.30×10^{-6}
17	hsa05168	Herpes simplex virus 1 infection	13	13/99	1.30×10^{-6}
18	hsa05212	Pancreatic cancer	9	9/99	1.98×10^{-6}
19	hsa04510	Focal adhesion	13	13/99	3.97×10^{-6}
20	hsa05169	Epstein-Barr virus infection	13	13/99	3.97×10^{-6}
