# Supplementary material for: Comparative Network Pharmacology and Molecular Docking Analysis of Ursodeoxycholic Acid and Tauroursodeoxycholic Acid in Alzheimer’s Disease-Associated Molecular Networks
Source: Pharmaceuticals (Basel). 2026 Sep 23;19(10):1512. doi: 10.3390/ph19101512 (PMC13708223; doi:10.3390/ph19101512)
Supplement: Supplementary file 1 [file pharmaceuticals-19-01512-s001.zip › Supplementary Table S4.pdf]

KEGG pathway enrichment results for UDCA-AD intersection targets

Rank	KEGG ID	Pathway	Count	GeneRatio	Adjusted p value
1	hsa04080	Neuroactive ligand-receptor interaction	23	23/90	7.87×10^{-11}
2	hsa04082	Neuroactive ligand signaling	15	15/90	5.73×10^{-8}
3	hsa05207	Chemical carcinogenesis - receptor activation	15	15/90	1.28×10^{-7}
4	hsa03320	PPAR signaling pathway	9	9/90	2.08×10^{-6}
5	hsa04659	Th17 cell differentiation	9	9/90	3.85×10^{-5}
6	hsa04081	Hormone signaling	12	12/90	3.85×10^{-5}
7	hsa04919	Thyroid hormone signaling pathway	9	9/90	7.11×10^{-5}
8	hsa04915	Estrogen signaling pathway	9	9/90	1.83×10^{-4}
9	hsa04148	Efferocytosis	9	9/90	4.36×10^{-4}
10	hsa05205	Proteoglycans in cancer	10	10/90	5.16×10^{-4}
11	hsa05222	Small cell lung cancer	7	7/90	5.62×10^{-4}
12	hsa00140	Steroid hormone biosynthesis	6	6/90	6.78×10^{-4}
13	hsa01522	Endocrine resistance	7	7/90	7.14×10^{-4}
14	hsa04920	Adipocytokine signaling pathway	6	6/90	8.13×10^{-4}
15	hsa04917	Prolactin signaling pathway	6	6/90	8.23×10^{-4}
16	hsa05223	Non-small cell lung cancer	6	6/90	9.03×10^{-4}
17	hsa04072	Phospholipase D signaling pathway	8	8/90	1.10×10^{-3}
18	hsa04724	Glutamatergic synapse	7	7/90	1.42×10^{-3}
19	hsa05412	Arrhythmogenic right ventricular cardiomyopathy	6	6/90	1.90×10^{-3}
20	hsa04611	Platelet activation	7	7/90	2.03×10^{-3}
