# Supplementary material for: Comparative Network Pharmacology and Molecular Docking Analysis of Ursodeoxycholic Acid and Tauroursodeoxycholic Acid in Alzheimer’s Disease-Associated Molecular Networks
Source: Pharmaceuticals (Basel). 2026 Sep 23;19(10):1512. doi: 10.3390/ph19101512 (PMC13708223; doi:10.3390/ph19101512)
Supplement: Supplementary file 1 [file pharmaceuticals-19-01512-s001.zip › Supplementary Table S5.pdf]

UDCA vs TUDCA

Dimension	UDCA	TUDCA	Comparative interpretation
Chemical relationship	Parent hydrophilic bile acid	Taurine-conjugated derivative of UDCA	The two compounds share the same steroidal bile acid backbone but differ at the side chain, where TUDCA contains an additional taurine-conjugated moiety.
Core structural feature	Free carboxyl group at the C24 side chain	Taurine-conjugated amide side chain with a sulfonic acid-containing polar tail	Taurine conjugation increases molecular polarity and hydrogen-bonding capacity, providing a plausible structural basis for the distinct pharmacokinetic and docking profiles of TUDCA.
Molecular weight	392.29	499.3	TUDCA is substantially larger than UDCA, which may contribute to reduced passive permeability but stronger polar interaction capacity.
TPSA	77.76 Å²	123.93 Å²	TUDCA shows markedly higher polarity, whereas UDCA retains a more absorption-favorable physicochemical profile.
logP	4.734	3.438	UDCA is more lipophilic, while TUDCA is comparatively more hydrophilic after taurine conjugation.
Hydrogen-bond acceptors / donors	nHA 4; nHD 3	nHA 7; nHD 4	TUDCA has greater hydrogen-bonding potential, which may favor polar interactions within protein pockets.
Predicted compound targets	106	111	Both bile acids showed a similar scale of predicted targets, suggesting broad multi-target potential.
AD-overlapping targets	98	103	Both compounds showed substantial intersection with AD-related genes, indicating partial mechanistic convergence.

Overall network tendency	More associated with nuclear receptor-linked and metabolic regulatory networks	More associated with intracellular signaling, survival regulation, and apoptosis-related networks	The two bile acids are not pharmacologically identical; instead, they show different dominant mechanistic emphases in the AD-related target landscape.
Representative specific targets / features	ESR1, PPARA, RXRA were more prominent	BCL2, JAK2, PIK3CA, MTOR, PTGS2 were more prominent	UDCA tends to align with receptor-mediated transcriptional/metabolic regulation, whereas TUDCA is more closely linked to kinase signaling and cell-survival control.
GO enrichment tendency	Enriched in nuclear receptor activity, ligand-activated transcription factor activity, and steroid binding	Enriched in kinase-related and receptor-mediated signaling functions, including protein serine/threonine kinase activity and phosphatase binding	GO analysis supports a more transcriptional-regulatory profile for UDCA and a more signaling-oriented profile for TUDCA.
KEGG enrichment tendency	More closely related to PPAR signaling, estrogen signaling, thyroid hormone signaling, and receptor-linked regulatory pathways	More closely related to PI3K–Akt, JAK–STAT, cAMP, neurotrophin, and inflammation-related pathways	KEGG analysis suggests that UDCA is biased toward receptor/hormone-associated regulation, whereas TUDCA is biased toward survival and stress-response signaling.
PPI hub characteristics	Hub nodes mainly included SRC, ESR1, EGFR, PPARA, MAPK14, RXRA, RARA, and AR	Hub nodes mainly included BCL2, MTOR, PTGS2, JAK2, and PIK3CA	The PPI networks further support the divergence between a nuclear receptor/metabolic axis for UDCA and a signaling/apoptosis axis for TUDCA.
Docking target preference	Preferential docking to nuclear receptor-related and	Preferential docking to kinase- and apoptosis-related proteins	Structural-level prediction is broadly consistent with the network pharmacology findings.

	transcription-associated targets		
Docking energy range	−8.1 to −7.0 kcal/mol	−10.3 to −7.4 kcal/mol	TUDCA showed lower predicted binding-energy values across selected prioritized targets.
Best docking target	PPARA (−8.1 kcal/mol)	PIK3CA (−10.3 kcal/mol)	The strongest predicted UDCA interaction was observed with a nuclear receptor-related target, whereas the strongest predicted TUDCA interaction was observed with a key signaling kinase target.
Representative docking interpretation	More compatible with nuclear receptor-linked regulatory binding pockets	More compatible with polar, signaling-related, or apoptosis-associated pockets	The docking profiles suggest distinct target-binding tendencies rather than a simple potency ranking between the two bile acids.
Caco-2 permeability	−5.386	−5.507	UDCA showed slightly more favorable predicted intestinal permeability.
HIA prediction	0.007	0.662	UDCA showed a more favorable predicted human intestinal absorption profile than TUDCA.
Oral bioavailability tendency	Lower predicted probability of poor oral bioavailability (F20%, F30%, F50%)	Higher predicted probability of poor oral bioavailability across F20%, F30%, and F50% models	UDCA appears more favorable for absorption-related behavior, while TUDCA may be disadvantaged by higher polarity and molecular size.
Plasma protein binding (PPB)	90.49%	59.86%	UDCA is predicted to be more extensively protein-bound, whereas TUDCA may circulate with a higher free fraction.
Fraction unbound (Fu)	8.80%	36.43%	TUDCA shows a substantially higher predicted free fraction, which may affect tissue availability and pharmacological exposure.

BBB prediction	0.116	0.059	Neither compound showed a clear BBB-positive prediction; UDCA was slightly higher, but the difference does not support a strong BBB advantage for TUDCA.
CYP inhibition profile	Overall low inhibition of major CYP isoforms; predicted CYP3A4 substrate probability 0.999	Overall low inhibition of major CYP isoforms; predicted CYP3A4 substrate probability 0.008	Both bile acids showed limited broad CYP inhibition liability, but UDCA may have a stronger association with CYP3A4-dependent metabolism.
HLM stability	0	0.011	Both compounds were predicted to be relatively stable in human liver microsomes.
Plasma clearance	6.221 mL/min/kg	12.636 mL/min/kg	TUDCA showed higher predicted plasma clearance than UDCA.
Predicted half-life	1.824 h	1.517 h	Both compounds were predicted to have short half-lives, with UDCA being slightly longer.
hERG risk	Low	Low	Neither compound showed a strong predicted hERG liability.
Ames mutagenicity	Low predicted risk	Low predicted risk	Neither compound showed a strong mutagenicity warning in silico.
DILI tendency	0.274	0.272	Predicted liver injury risk was similar between the two compounds.
Skin sensitization	0.939	0.994	Both compounds showed a notable skin sensitization warning in silico, with TUDCA slightly higher.
Nephrotoxicity warning	0.712	0.93	Both compounds showed predicted nephrotoxicity warning signals, with TUDCA appearing higher.

Overall comparative profile	More consistent with a receptor-regulatory and metabolism-oriented intervention profile	More consistent with a survival-signaling and anti-apoptotic intervention profile	Taken together, UDCA and TUDCA appear partially overlapping but mechanistically non-equivalent, supporting their comparison as distinct rather than interchangeable bile acid candidates in AD-related research.
