# Supplementary material for: Comparative Network Pharmacology and Molecular Docking Analysis of Ursodeoxycholic Acid and Tauroursodeoxycholic Acid in Alzheimer’s Disease-Associated Molecular Networks
Source: Pharmaceuticals (Basel). 2026 Sep 23;19(10):1512. doi: 10.3390/ph19101512 (PMC13708223; doi:10.3390/ph19101512)
Supplement: Supplementary file 1 [file pharmaceuticals-19-01512-s001.zip › Supplementary Table S6.pdf]

Redocking RMSD values

Target Protein	PDB ID	Native Ligand	RMSD(Å)
CYP19A1	3S79	ASD	1.790
EGFR	4WKQ	IRE	1.971
ESR1	2IOG	IOG	0.239
PPARA	6KAX	PLM	0.059
RXRA	5MKU	J57	0.063
SRC	1YOL	S03	0.360
BCL2	8HTS	N2L	0.941
JAK2	3UGC	046	2.169
KIT	1T46	STI	0.611
MTOR	4DRI	RAP	3.872
PIK3CA	7JIU	LOI	0.990
PTGS2	3LN1	CEL	0.891
MAPK14	4R3C	3GF	0.239
GPBAR1	7CFN	INT-777	0.000
