# Supplementary material for: Comparative Network Pharmacology and Molecular Docking Analysis of Ursodeoxycholic Acid and Tauroursodeoxycholic Acid in Alzheimer’s Disease-Associated Molecular Networks
Source: Pharmaceuticals (Basel). 2026 Sep 23;19(10):1512. doi: 10.3390/ph19101512 (PMC13708223; doi:10.3390/ph19101512)
Supplement: Supplementary file 1 [file pharmaceuticals-19-01512-s001.zip › Supplementary Table S7.pdf]

Network topology analysis of UDCA-related targets and selection of candidate targets for molecular docking

**Target**	**Degree**	**Degree Rank**	**Betweenness Centrality**	**Closeness Centrality**	**Selected for Docking**
SRC	44	1	2136.737	0.007042	Yes
ESR1	32	2	1248.022	0.006667	Yes
EGFR	32	2	899.825	0.006410	Yes
PPARA	25	4	702.615	0.005952	Yes
RXRA	21	5	278.891	0.005747	Yes
CYP19A1	17	6	459.498	0.005714	Yes
RARA	17	6	230.325	0.005435	No
NR0B2	17	6	148.494	0.005587	No
HMGCR	16	9	525.307	0.005208	No
PTPRC	16	9	104.546	0.005376	No
MMP2	16	9	76.367	0.005405	No
PPARD	15	12	113.888	0.005102	No
GRM5	14	13	263.594	0.005181	No
ESR2	14	13	143.780	0.005618	No
PTPN1	14	13	109.095	0.005405	No
NR1H4	14	13	86.772	0.005051	No
AR	13	17	135.143	0.005495	No
RXRG	13	17	36.714	0.004464	No
ITGB1	13	17	23.967	0.005102	No
