# Supplementary material for: Comparative Network Pharmacology and Molecular Docking Analysis of Ursodeoxycholic Acid and Tauroursodeoxycholic Acid in Alzheimer’s Disease-Associated Molecular Networks
Source: Pharmaceuticals (Basel). 2026 Sep 23;19(10):1512. doi: 10.3390/ph19101512 (PMC13708223; doi:10.3390/ph19101512)
Supplement: Supplementary file 1 [file pharmaceuticals-19-01512-s001.zip › Supplementary Table S8.pdf]

Network topology analysis of TUDCA-related targets and selection of candidate targets for molecular docking

Target	Degree	Degree Rank	Betweenness Centrality	Closeness Centrality	Selected for Docking
BCL2	70	1	1245.954	0.005525	Yes
PTGS2	62	2	972.726	0.005319	Yes
MTOR	62	2	689.414	0.005181	Yes
KIT	50	4	586.406	0.005181	Yes
JAK2	50	4	374.640	0.004902	Yes
PIK3CA	48	6	199.053	0.004484	Yes
BCL2L1	44	7	169.738	0.004854	No
MDM2	40	8	312.356	0.004739	No
ABCB1	36	9	816.280	0.005236	No
SLC6A4	36	9	605.717	0.004525	No
OPRM1	32	11	386.851	0.004405	No
DRD2	30	12	295.169	0.004464	No
CYP3A4	28	13	488.207	0.005000	No
ITGB3	28	13	467.691	0.004808	No
SLC6A3	28	13	208.570	0.004065	No
MME	24	16	484.898	0.004651	No
ITGA2B	24	16	301.575	0.004386	No
PSEN1	24	16	274.498	0.004386	No
CYP2C9	24	16	263.891	0.004484	No
ACHE	22	20	607.432	0.004902	No
HMGCR	22	20	194.901	0.004695	No
SIGMAR1	20	22	214.147	0.004525	No
