# Supplementary material for: Comparative Network Pharmacology and Molecular Docking Analysis of Ursodeoxycholic Acid and Tauroursodeoxycholic Acid in Alzheimer’s Disease-Associated Molecular Networks
Source: Pharmaceuticals (Basel). 2026 Sep 23;19(10):1512. doi: 10.3390/ph19101512 (PMC13708223; doi:10.3390/ph19101512)
Supplement: Supplementary file 1 [file pharmaceuticals-19-01512-s001.zip › Supplementary Table S9.pdf]

Effect of different GeneCards relevance score thresholds on AD-associated targets and drug-disease intersections

**GeneCards relevance score threshold**	**GeneCards genes (merged from 3 keywords)**	**OMIM genes**	**Final AD-related genes after merging**	**UDCA–AD overlapping targets**	**TUDCA–AD overlapping targets**
>0	17,205	221	17,215	98	103
>1	13,632	221	13,645	98	103
>5	6,390	221	6,426	81	99
>10	3,718	221	3,767	61	77
