# Supplementary material for: Comparative Network Pharmacology and Molecular Docking Analysis of Ursodeoxycholic Acid and Tauroursodeoxycholic Acid in Alzheimer’s Disease-Associated Molecular Networks
Source: Pharmaceuticals (Basel). 2026 Sep 23;19(10):1512. doi: 10.3390/ph19101512 (PMC13708223; doi:10.3390/ph19101512)
Supplement: Supplementary file 1 [file pharmaceuticals-19-01512-s001.zip › Supplementary Table S10.pdf]

Stability of UDCA-associated hub targets under different GeneCards relevance score thresholds

Compound	Hub target	>0	>1	>5	>10
UDCA	PPARA	+	+	+	+
UDCA	ESR1	+	+	+	+
UDCA	RXRA	+	+	+	+
UDCA	CYP19A1	+	+	+	+
UDCA	EGFR	+	+	+	+
UDCA	SRC	+	+	+	+
