# Supplementary material for: Comparative Network Pharmacology and Molecular Docking Analysis of Ursodeoxycholic Acid and Tauroursodeoxycholic Acid in Alzheimer’s Disease-Associated Molecular Networks
Source: Pharmaceuticals (Basel). 2026 Sep 23;19(10):1512. doi: 10.3390/ph19101512 (PMC13708223; doi:10.3390/ph19101512)
Supplement: Supplementary file 1 [file pharmaceuticals-19-01512-s001.zip › Supplementary Table S11.pdf]

Stability of TUDCA-associated hub targets under different GeneCards relevance score thresholds

**Compound**	**Hub target**	**>0**	**>1**	**>5**	**>10**
TUDCA	BCL2	+	+	+	+
TUDCA	MTOR	+	+	+	+
TUDCA	PTGS2	+	+	+	+
TUDCA	JAK2	+	+	+	+
TUDCA	PIK3CA	+	+	+	+
