# Supplementary material for: Comparative Network Pharmacology and Molecular Docking Analysis of Ursodeoxycholic Acid and Tauroursodeoxycholic Acid in Alzheimer’s Disease-Associated Molecular Networks
Source: Pharmaceuticals (Basel). 2026 Sep 23;19(10):1512. doi: 10.3390/ph19101512 (PMC13708223; doi:10.3390/ph19101512)
Supplement: Supplementary file 1 [file pharmaceuticals-19-01512-s001.zip › Supplementary Table S12.pdf]

Summary of independent target support

Compound	Predicted targets (SwissTargetPrediction)	DrugBank-supported targets	ChEMBL-supported targets	Independently supported targets
UDCA	100	1	9	9
TUDCA	100	0*	2	2
