# Supplementary material for: Comparative Network Pharmacology and Molecular Docking Analysis of Ursodeoxycholic Acid and Tauroursodeoxycholic Acid in Alzheimer’s Disease-Associated Molecular Networks
Source: Pharmaceuticals (Basel). 2026 Sep 23;19(10):1512. doi: 10.3390/ph19101512 (PMC13708223; doi:10.3390/ph19101512)
Supplement: Supplementary file 1 [file pharmaceuticals-19-01512-s001.zip › Supplementary Table S13.pdf]

Independent evidence supporting UDCA-associated key targets

Target	UniProt ID	Role in current study	SwissTargetPrediction	DrugBank	ChEMBL
GPBAR1	Q8TDU6	Shared bile acid receptor; selected for same-target docking and MD	Yes	No	Yes
NR1H4	Q96RI1	Canonical bile acid receptor (FXR)	Yes	Yes	Yes
ESR1	P03372	UDCA PPI hub target	Yes	No	Yes
PPARA	P37231	UDCA nuclear receptor-associated hub target	Yes	No	Not identified in current ChEMBL export
RXRA	P19793	UDCA nuclear receptor-associated target	Yes	No	Not identified in current ChEMBL export
SRC	P12931	Highest-degree UDCA PPI hub	Yes	No	Not identified in current ChEMBL export
CYP19A1	P11511	UDCA docking target	Yes	No	Not identified in current ChEMBL export
