# Supplementary material for: Comparative Network Pharmacology and Molecular Docking Analysis of Ursodeoxycholic Acid and Tauroursodeoxycholic Acid in Alzheimer’s Disease-Associated Molecular Networks
Source: Pharmaceuticals (Basel). 2026 Sep 23;19(10):1512. doi: 10.3390/ph19101512 (PMC13708223; doi:10.3390/ph19101512)
Supplement: Supplementary file 1 [file pharmaceuticals-19-01512-s001.zip › Supplementary Table S14.pdf]

Predicted targets of ursodeoxycholic acid (UDCA) based on ChEMBL database analysis

**Target**	**Common name**	**Uniprot ID**	**ChEMBL ID**	**Target Class**	**Probability***	**Known actives (3D/2D)**
G-protein coupled bile acid receptor 1	GPBAR1	Q8TDU6	CHEMBL5409	Family A G protein-coupled receptor	0.90726583922	22 / 16
Bile acid receptor FXR	NR1H4	Q96RI1	CHEMBL2047	Nuclear receptor	0.88832702124	15 / 12
Vitamin D receptor	VDR	P11473	CHEMBL1977	Nuclear receptor	0.615836016661	8 / 3
DNA polymerase beta (by homology)	POLB	P06746	CHEMBL2392	Enzyme	0.591260490229	2 / 5
Aldo-keto reductase family 1 member B10	AKR1B10	O60218	CHEMBL5983	Enzyme	0.574895354504	4 / 7
Androgen Receptor	AR	P10275	CHEMBL1871	Nuclear receptor	0.435493313062	6 / 47
Dual specificity phosphatase Cdc25A	CDC25A	P30304	CHEMBL3775	Phosphatase	0.279715408293	0 / 14
11-beta-hydroxysteroid dehydrogenase 1	HSD11B1	P28845	CHEMBL4235	Enzyme	0.197714623027	17 / 30
UDP-glucuronosyltransferase 2B7	UGT2B7	P16662	CHEMBL4370	Enzyme	0.181322641621	0 / 26
Corticosteroid binding globulin	SERPINA6	P08185	CHEMBL2421	Secreted protein	0.181322641621	0 / 11
Testis-specific androgen-binding protein	SHBG	P04278	CHEMBL3305	Secreted protein	0.181322641621	0 / 31

Estradiol 17-beta-dehydrogenase 3	HSD17B3	P37058	CHEMBL4234	Enzyme	0.181322641621	0 / 13
Glucose-6-phosphate 1-dehydrogenase	G6PD	P11413	CHEMBL5347	Enzyme	0.181322641621	0 / 10
GABA-B receptor (by homology)	GABBR1	Q9UBS5	CHEMBL2064	Family C G protein-coupled receptor	0.181322641621	0 / 1
Cytochrome P450 19A1	CYP19A1	P11511	CHEMBL1978	Cytochrome P450	0.173117202483	1 / 33
Prostanoid EP2 receptor	PTGER2	P43116	CHEMBL1881	Family A G protein-coupled receptor	0.148531769662	114 / 7
Prostanoid FP receptor	PTGFR	P43088	CHEMBL1987	Family A G protein-coupled receptor	0.148531769662	22 / 2
Niemann-Pick C1-like protein 1	NPC1L1	Q9UHC9	CHEMBL2027	Other membrane protein	0.140333097536	0 / 6
GABA A receptor alpha-2/beta-2/gamma-2	GABRA2 GABRB2 GABRG2	P47869 P47870 P18507	CHEMBL2111413	Ligand-gated ion channel	0.140333097536	0 / 1
Fatty acid binding protein muscle	FABP3	P05413	CHEMBL3344	Fatty acid binding protein family	0.140333097536	0 / 5
Fatty acid binding protein epidermal	FABP5	Q01469	CHEMBL3674	Fatty acid binding protein family	0.140333097536	0 / 2

Fatty acid binding protein intestinal	FABP2	P12104	CHEMBL4879	Fatty acid binding protein family	0.140333097536	0 / 1
Peroxisome proliferator-activated receptor alpha	PPARA	Q07869	CHEMBL239	Nuclear receptor	0.132134815861	11 / 10
Peroxisome proliferator-activated receptor delta	PPARD	Q03181	CHEMBL3979	Nuclear receptor	0.132134815861	16 / 8
Protein farnesyltransferase	FNTA FNTB	P49354 P49356	CHEMBL2094108	Enzyme	0.11573667475	12 / 8
11-beta-hydroxysteroid dehydrogenase 2	HSD11B2	P80365	CHEMBL3746	Enzyme	0.11573667475	6 / 5
Fatty acid binding protein adipocyte	FABP4	P15090	CHEMBL2083	Fatty acid binding protein family	0.11573667475	3 / 3
Free fatty acid receptor 1	FFAR1	O14842	CHEMBL4422	Family A G protein-coupled receptor	0.11573667475	1 / 3
Lysine-specific demethylase 2A	KDM2A	Q9Y2K7	CHEMBL1938210	Eraser	0.11573667475	0 / 1
Histone lysine demethylase PHF8	PHF8	Q9UPP1	CHEMBL1938212	Eraser	0.11573667475	0 / 1
Lysine-specific demethylase 5C	KDM5C	P41229	CHEMBL2163176	Eraser	0.11573667475	0 / 1

Solute carrier family 22 member 6 (by homology)	SLC22A6	Q4U2R8	CHEMBL1641347	Electrochemical transporter	0.11573667475	1 / 2
CDC45-related protein	CDC45	O75419	CHEMBL3040	Other nuclear protein	0.11573667475	0 / 1
Leukocyte common antigen	PTPRC	P08575	CHEMBL3243	Enzyme	0.11573667475	0 / 1
Protein-tyrosine phosphatase 1B	PTPN1	P18031	CHEMBL335	Phosphatase	0.11573667475	36 / 27
Plasminogen	PLG	P00747	CHEMBL1801	Protease	0.11573667475	0 / 1
Hydroxyacid oxidase 1	HAO1	Q9UJM8	CHEMBL4229	Enzyme	0.11573667475	0 / 1
Prostanoid DP receptor	PTGDR	Q13258	CHEMBL4427	Family A G protein-coupled receptor	0.11573667475	44 / 0
Thyroid hormone receptor alpha	THRA	P10827	CHEMBL1860	Nuclear receptor	0.11573667475	64 / 0
Thyroid hormone receptor beta-1	THRB	P10828	CHEMBL1947	Nuclear receptor	0.11573667475	72 / 0
Thromboxane A2 receptor	TBXA2R	P21731	CHEMBL2069	Family A G protein-coupled receptor	0.11573667475	50 / 0
LXR-alpha	NR1H3	Q13133	CHEMBL2808	Nuclear receptor	0.11573667475	5 / 6
Prostanoid EP4 receptor (by homology)	PTGER4	P35408	CHEMBL1836	Family A G protein-coupled receptor	0.11573667475	165 / 0
Retinoic acid receptor gamma	RARG	P13631	CHEMBL2003	Nuclear receptor	0.11573667475	21 / 0

Retinoic acid receptor beta	RARB	P10826	CHEMBL2008	Nuclear receptor	0.11573667475	29 / 0
Voltage-gated calcium channel alpha2/delta subunit 1	CACNA2D1	P54289	CHEMBL1919	Calcium channel auxiliary subunit alpha2delta family	0.11573667475	0 / 4
Betaine transporter	SLC6A12	P48065	CHEMBL3715	Electrochemical transporter	0.11573667475	0 / 3
Glutathione S-transferase kappa 1	GSTK1	Q9Y2Q3	CHEMBL4491	Enzyme	0.11573667475	0 / 1
Neuronal acetylcholine receptor protein alpha-7 subunit (by homology)	CHRNA7	P36544	CHEMBL2492	Ligand-gated ion channel	0.11573667475	0 / 2
Tyrosine-protein kinase JAK3	JAK3	P52333	CHEMBL2148	Kinase	0.11573667475	3 / 0
Glutamate carboxypeptidase II	FOLH1	Q04609	CHEMBL1892	Protease	0.0	0 / 10
Metabotropic glutamate receptor 5	GRM5	P41594	CHEMBL3227	Family C G protein-coupled receptor	0.0	0 / 4
Retinoic acid receptor alpha	RARA	P10276	CHEMBL2055	Nuclear receptor	0.0	21 / 0
Estrogen receptor alpha	ESR1	P03372	CHEMBL206	Nuclear receptor	0.0	100 / 1
Integrin alpha-IIb/beta-3	ITGA2B ITGB3	P08514 P05106	CHEMBL2093869	Membrane receptor	0.0	119 / 0

Dual specificity phosphatase Cdc25B	CDC25B	P30305	CHEMBL4804	Phosphatase	0.0	0 / 6
Nuclear receptor subfamily 1 group I member 3	NR1I3	Q14994	CHEMBL5503	Nuclear receptor	0.0	0 / 2
Tyrosine-protein kinase JAK1	JAK1	P23458	CHEMBL2835	Kinase	0.0	3 / 0
Tyrosine-protein kinase JAK2	JAK2	O60674	CHEMBL2971	Kinase	0.0	1 / 0
HMG-CoA reductase	HMGCR	P04035	CHEMBL402	Oxidoreductase	0.0	117 / 21
Cytosolic phospholipase A2	PLA2G4A	P47712	CHEMBL3816	Enzyme	0.0	29 / 0
Bile acid transporter	SLC10A1	Q14973	CHEMBL5287	Electrochemical transporter	0.0	2 / 0
SUMO-activating enzyme	SAE1 UBA2	Q9UBE0 Q9UBT2	CHEMBL2095174	Enzyme	0.0	1 / 1
Nuclear receptor subfamily 0 group B member 2	NR0B2	Q15466	CHEMBL5603	Nuclear receptor	0.0	3 / 0
Prostaglandin E synthase	PTGES	O14684	CHEMBL5658	Enzyme	0.0	2 / 8
Estrogen receptor beta	ESR2	Q92731	CHEMBL242	Nuclear receptor	0.0	16 / 6
Prostanoid EP3 receptor	PTGER3	P43115	CHEMBL3710	Family A G protein-coupled receptor	0.0	28 / 0
Carbonic anhydrase II	CA2	P00918	CHEMBL205	Lyase	0.0	2 / 4
Retinoid X receptor alpha	RXRA	P19793	CHEMBL2061	Nuclear receptor	0.0	18 / 0

Bromodomain-containing protein 4	BRD4	O60885	CHEMBL1163125	Reader	0.0	2 / 0
Retinoid X receptor beta	RXRB	P28702	CHEMBL1870	Nuclear receptor	0.0	10 / 0
Retinoid X receptor gamma	RXRG	P48443	CHEMBL2004	Nuclear receptor	0.0	13 / 0
Epidermal growth factor receptor erbB1	EGFR	P00533	CHEMBL203	Kinase	0.0	4 / 0
Tyrosine-protein kinase SRC	SRC	P12931	CHEMBL267	Kinase	0.0	4 / 0
Matrix metalloproteinase 3	MMP3	P08254	CHEMBL283	Protease	0.0	5 / 0
Matrix metalloproteinase 1	MMP1	P03956	CHEMBL332	Protease	0.0	4 / 0
Matrix metalloproteinase 2	MMP2	P08253	CHEMBL333	Protease	0.0	6 / 0
MAP kinase p38 alpha	MAPK14	Q16539	CHEMBL260	Kinase	0.0	7 / 0
c-Jun N-terminal kinase 3	MAPK10	P53779	CHEMBL2637	Kinase	0.0	5 / 0
Metabotropic glutamate receptor 3	GRM3	Q14832	CHEMBL2888	Family C G protein-coupled receptor	0.0	0 / 11
Metabotropic glutamate receptor 1	GRM1	Q13255	CHEMBL3772	Family C G protein-coupled receptor	0.0	0 / 4
Metabotropic glutamate receptor 6	GRM6	O15303	CHEMBL4573	Family C G protein-coupled receptor	0.0	0 / 8

Metabotropic glutamate receptor 2	GRM2	Q14416	CHEMBL5137	Family C G protein-coupled receptor	0.0	0 / 19
Integrin alpha-V/beta-3	ITGAV ITGB3	P06756 P05106	CHEMBL1907598	Membrane receptor	0.0	65 / 0
Integrin alpha-5/beta-1	ITGB1 ITGA5	P05556 P08648	CHEMBL2095226	Membrane receptor	0.0	1 / 0
Sonic hedgehog protein (by homology)	SHH	Q15465	CHEMBL5602	Unclassified protein	0.0	0 / 10
Cholecystokinin B receptor (by homology)	CCKBR	P32239	CHEMBL298	Family A G protein-coupled receptor	0.0	22 / 0
Xaa-Pro dipeptidase	PEPD	P12955	CHEMBL4185	Protease	0.0	0 / 1
Mu opioid receptor	OPRM1	P35372	CHEMBL233	Family A G protein-coupled receptor	0.0	39 / 0
Delta opioid receptor	OPRD1	P41143	CHEMBL236	Family A G protein-coupled receptor	0.0	36 / 0
Kappa Opioid receptor	OPRK1	P41145	CHEMBL237	Family A G protein-coupled receptor	0.0	19 / 0
T-cell protein-tyrosine phosphatase	PTPN2	P17706	CHEMBL3807	Phosphatase	0.0	3 / 13
WD repeat-containing protein 5	WDR5	P61964	CHEMBL1075317	Unclassified protein	0.0	2 / 0
Aldo-keto-reductase family 1 member C3	AKR1C3	P42330	CHEMBL4681	Enzyme	0.0	10 / 0

Excitatory amino acid transporter 2	SLC1A2	P43004	CHEMBL4973	Electrochemical transporter	0.0	0 / 3
Calcium sensing receptor	CASR	P41180	CHEMBL1878	Family C G protein-coupled receptor	0.0	4 / 0
Dipeptidyl peptidase IV	DPP4	P27487	CHEMBL284	Protease	0.0	49 / 0
Renin	REN	P00797	CHEMBL286	Protease	0.0	3 / 0
Acyl coenzyme A:cholesterol acyltransferase 1	SOAT1	P35610	CHEMBL2782	Enzyme	0.0	2 / 0
Diacylglycerol O-acyltransferase 1	DGAT1	O75907	CHEMBL6009	Enzyme	0.0	35 / 0
