# Supplementary material for: Comparative Network Pharmacology and Molecular Docking Analysis of Ursodeoxycholic Acid and Tauroursodeoxycholic Acid in Alzheimer’s Disease-Associated Molecular Networks
Source: Pharmaceuticals (Basel). 2026 Sep 23;19(10):1512. doi: 10.3390/ph19101512 (PMC13708223; doi:10.3390/ph19101512)
Supplement: Supplementary file 1 [file pharmaceuticals-19-01512-s001.zip › Supplementary Table S1.pdf]

GO molecular function enrichment results for UDCA-AD intersection targets

Rank	GO ID	Molecular function term	Count	GeneRatio	Adjusted p value
1	GO:0004879	nuclear receptor activity	17	17/98	2.57×10^{-23}
2	GO:0098531	ligand-activated transcription factor activity	17	17/98	2.57×10^{-23}
3	GO:0005496	steroid binding	12	12/98	5.39×10^{-11}
4	GO:0004954	prostanoid receptor activity	6	6/98	8.29×10^{-10}
5	GO:0003707	nuclear steroid receptor activity	7	7/98	3.20×10^{-9}
6	GO:0004953	icosanoid receptor activity	6	6/98	5.88×10^{-9}
7	GO:0004955	prostaglandin receptor activity	5	5/98	5.20×10^{-8}
8	GO:0033293	monocarboxylic acid binding	8	8/98	4.21×10^{-7}
9	GO:0046965	nuclear retinoid X receptor binding	5	5/98	9.65×10^{-7}
10	GO:0098960	postsynaptic neurotransmitter receptor activity	7	7/98	2.98×10^{-6}
11	GO:0001223	transcription coactivator binding	6	6/98	4.60×10^{-6}
12	GO:0042974	nuclear retinoic acid receptor binding	5	5/98	5.75×10^{-6}
13	GO:0001221	transcription coregulator binding	8	8/98	5.75×10^{-6}
14	GO:0008066	glutamate receptor activity	5	5/98	6.36×10^{-6}
15	GO:0016922	nuclear receptor binding	8	8/98	1.68×10^{-5}
16	GO:0030594	neurotransmitter receptor activity	7	7/98	1.97×10^{-5}
17	GO:0005178	integrin binding	8	8/98	4.90×10^{-5}
18	GO:0061629	RNA polymerase II-specific DNA-binding transcription factor binding	11	11/98	4.90×10^{-5}
19	GO:0016616	oxidoreductase activity, acting on the CH-OH group of donors, NAD or NADP as acceptor	7	7/98	7.49×10^{-5}
20	GO:0001618	virus receptor activity	6	6/98	7.49×10^{-5}
