# Supplementary material for: Comparative Network Pharmacology and Molecular Docking Analysis of Ursodeoxycholic Acid and Tauroursodeoxycholic Acid in Alzheimer’s Disease-Associated Molecular Networks
Source: Pharmaceuticals (Basel). 2026 Sep 23;19(10):1512. doi: 10.3390/ph19101512 (PMC13708223; doi:10.3390/ph19101512)
Supplement: Supplementary file 1 [file pharmaceuticals-19-01512-s001.zip › Supplementary Table S2.pdf]

GO molecular function enrichment results for TUDCA-AD intersection targets

Rank	GO ID	Molecular function term	Count	GeneRatio	Adjusted p value
1	GO:0106310	protein serine kinase activity	18	18/103	4.31×10^{-10}
2	GO:0008528	G protein-coupled peptide receptor activity	12	12/103	8.26×10^{-10}
3	GO:0001653	peptide receptor activity	12	12/103	9.60×10^{-10}
4	GO:0004674	protein serine/threonine kinase activity	18	18/103	1.74×10^{-9}
5	GO:0035173	histone kinase activity	13	13/103	1.11×10^{-7}
6	GO:0019903	protein phosphatase binding	10	10/103	4.05×10^{-7}
7	GO:0019902	phosphatase binding	11	11/103	4.21×10^{-7}
8	GO:0008227	G protein-coupled amine receptor activity	6	6/103	3.97×10^{-6}
9	GO:0044024	histone H2AS1 kinase activity	11	11/103	3.97×10^{-6}
10	GO:0140995	histone H2A kinase activity	11	11/103	3.97×10^{-6}
11	GO:0140993	histone modifying activity	14	14/103	4.02×10^{-6}
12	GO:0016303	1-phosphatidylinositol-3-kinase activity	4	4/103	5.30×10^{-6}
13	GO:0004993	G protein-coupled serotonin receptor activity	4	4/103	1.65×10^{-5}
14	GO:0004713	protein tyrosine kinase activity	8	8/103	2.38×10^{-5}
15	GO:0004089	carbonate dehydratase activity	4	4/103	2.38×10^{-5}
16	GO:0043176	amine binding	4	4/103	2.38×10^{-5}
17	GO:0099589	serotonin receptor activity	4	4/103	2.38×10^{-5}
18	GO:0004707	MAP kinase activity	4	4/103	2.99×10^{-5}
19	GO:0004712	protein serine/threonine/tyrosine kinase activity	5	5/103	7.04×10^{-5}
20	GO:0061133	endopeptidase activator activity	4	4/103	7.04×10^{-5}
